# A physicochemical double-cross-linked gelatin hydrogel with enhanced antibacterial and anti-inflammatory capabilities for improving wound healing

**DOI:** 10.1186/s12951-022-01634-z

**Published:** 2022-09-24

**Authors:** Yapeng Lu, Meihui Zhao, Ye Peng, Sizhe He, Xiaopeng Zhu, Chao Hu, Guanghua Xia, Tao Zuo, Xueying Zhang, Yonghuan Yun, Weimin Zhang, Xuanri Shen

**Affiliations:** 1 Hainan Engineering Research Center of Aquatic Resources Efficient Utilization in South China Sea, Key Laboratory of Food Nutrition and Functional Food of Hainan Province, Key Laboratory of Seafood Processing of Haikou, College of Food Science and Technology, Hainan University, Hainan, 570228 China; 2 Faculty of Medicine, Macau University of Science and Technology, Taipa, Macao SAR, China; 3 Collaborative Innovation Center of Provincial and Ministerial Co-Construction for Marine Food Deep Processing, Dalian Polytechnic University, Dalian, 116034 China; 4 Guangdong Institute of Gastroenterology, The Sixth Affiliated Hospital of Sun Yat-Sen University, Sun Yat-Sen University, Guangzhou, 510000 China

**Keywords:** Tilapia skin gelatin, Hydrogels, Anti-inflammatory, Wound microbiology, Wound healing

## Abstract

**Background:**

Skin tissue is vital in protecting the body from injuries and bacterial infections. Wound infection caused by bacterial colonization is one of the main factors hindering wound healing. Wound infection caused by colonization of a large number of bacteria can cause the wound to enter a continuous stage of inflammation, which delays wound healing. Hydrogel wound dressing is composed of natural and synthetic polymers, which can absorb tissue fluid, improve the local microenvironment of wound, and promote wound healing. However, in the preparation process of hydrogel, the complex preparation process and poor biological efficacy limit the application of hydrogel wound dressing in complex wound environment. Therefore, it is particularly important to develop and prepare hydrogel dressings with simple technology, good physical properties and biological effects by using natural polymers.

**Results:**

In this study, a gelatin-based (Tsg-THA&Fe) hydrogel was created by mixing trivalent iron (Fe^3+^) and 2,3,4-trihydroxybenzaldehyde (THA) to form a complex (THA&Fe), followed by a simple Schiff base reaction with tilapia skin gelatin (Tsg). The gel time and rheological properties of the hydrogels were adjusted by controlling the number of complexes. The dynamic cross-linking of the coordination bonds (o-phthalmictriol-Fe^3+^) and Schiff base bonds allows hydrogels to have good self-healing and injectable properties. In vitro experiments confirmed that the hydrogel had good biocompatibility and biodegradability as well as adhesion, hemostasis, and antibacterial properties. The feasibility of Tsg-THA&Fe hydrogel was studied by treating rat skin trauma model. The results showed that compared with Comfeel^®^ Plus Transparent dressing, the Tsg-THA&Fe hydrogel could obvious reduce the number of microorganisms, prevent bacterial colonization, reduce inflammation and accelerate wound healing. Local distribution of the Tsg-THA&Fe hydrogel in the skin tissue did not cause organ toxicity.

**Conclusions:**

In summary, the preparation process of Tsg-THA&Fe hydrogel is simple, with excellent performance in physical properties and biological efficacy. It can effectively relieve inflammation and control the colonization of wound microbes, and can be used as a multi-functional dressing to improve wound healing.

**Graphical Abstract:**

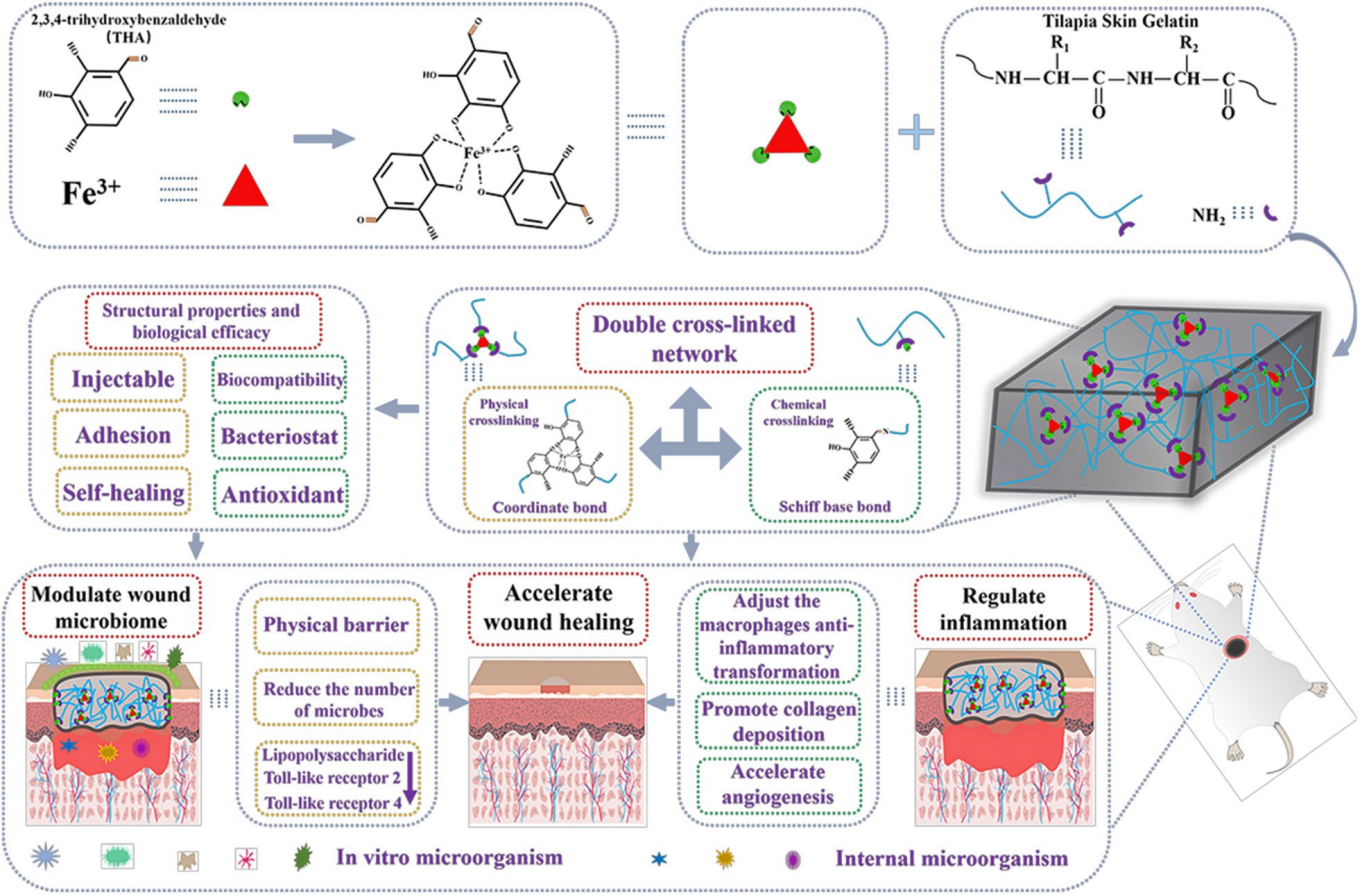

**Supplementary Information:**

The online version contains supplementary material available at 10.1186/s12951-022-01634-z.

## Introduction

Skin tissue integrity is critical for protecting the body from trauma and bacterial infection. The formation of irregular and infected wounds can significantly reduce the quality of healing. Wound problems have become medical problems that can threaten healthy lives [1–3]. The skin healing process consists of four major dynamic phases: hemostasis, inflammatory response, epidermal proliferation, and remodeling [4]. Various wound dressings have been developed to promote the healing environment. Hydrogel-based wound dressings are composed of a three-dimensional network formed by biocompatible materials that maintain a moderate healing environment, absorb tissue fluids, improve the local microenvironment of the wound, and promote wound healing. It is considered to be the most effective wound healing material [5–7]. To date, a wide variety of hydrogel dressings have been prepared using synthetic or natural polymers as raw materials, by means of chemical, physical, and enzymatic cross-linking [8–10]. However, in the preparation of hydrogels, the complex chemical modification of matrix materials and the use of special chemical cross-linking agents may greatly limit the commercial production and application of hydrogels [11, 12]. Hydrogel dressings make use of chemically synthesized matrix materials, such as polyvinyl alcohol, in the process of formation. Although there have been improvements in the physical properties of hydrogel dressings, they show poor biological efficacy without loaded functional factors, which makes them unsuitable for application to complex wounds [13, 14]. Therefore, it is particularly important to develop hydrogel dressings with a simple preparation process, good physical properties, and biological effects, using natural polymers. Gelatin-based hydrogels have been widely studied and applied in spite of their good biocompatibility and biodegradability [15, 16]. However, due to the complex preparation process of gelatin-based hydrogels, its superior biological activity function cannot be better demonstrated. Therefore, it is of great significance to study more stable and effective gelatin-based hydrogel dressings.

Injectable self-healing hydrogels formed using a hydrogel structural design through physical and chemical cross-linking exhibit excellent mechanical, self-healing, and adhesion properties, as well as biological efficacy, and are thus a hot topic in trauma dressing research [11, 17]. Many multifunctional hydrogels have been developed by taking inspiration from the excellent adhesion properties of mussels. Owing to the hydroxyl groups contained in catechol and o-triphenol, the hydrogel developed using these exhibits good adhesion properties [18, 19]. The self-healing properties of hydrogels are mainly achieved through dynamic physical and chemical interactions, where the phenolic hydroxyl groups of catechol and o-phenylene triol compounds can form coordination bonds with Fe^3+^, to achieve dynamic cross-linking of non-covalent bonds [17]. The reversible Schiff base bond is a dynamic chemical covalent bond that is commonly used to construct self-healing hydrogels [20–23]. The physicochemical double-cross-linked hydrogels constructed on the basis of Schiff base and phenoligylxyl-Fe^3+^ coordination bonds display accelerated gelation time as well as enhanced mechanical, tissue-adhesive, and self-healing properties, thereby making them more suitable for applications in complex environments [24–27].

Exposure of the wound site to the external environment and moist conditions resulting from the wound exudate leads to the formation of an extremely favorable environment for bacterial colonization of the wound site. Wound infection caused by the colonization of large numbers of bacteria is one of the main factors that prevents wound healing [28, 29]. Heavy bacterial colonization of the wound and production of endotoxins can cause a transition in the production of inflammatory factors, such as lipopolysaccharide (LPS). This pushes the wound into a sustained inflammatory phase, thereby delaying the healing process [30]. The immune response generated by the host through its immune cells has a protective effect on the wound and is important for the control of infection and wound healing at the wound site [31]. Therefore, synergistic application of treatments that reduce bacterial colonization and modulate inflammation could help improve wound healing. To achieve this therapeutic strategy, various anti-microbial agents have been applied in the production of hydrogel dressings, among which the main agents are antibiotic-based antimicrobial hydrogels [32–34]. The antibiotic class of drugs has a good antibacterial effect, but the resulting resistance hinders their application. Therefore, hydrogels with intrinsic antibacterial properties are the best choice for reducing bacterial contamination and inflammatory reactions, thereby enhancing wound healing.

In this study, we designed a physicochemical dual-cross-linked gelatin-based (Tsg-THA&Fe) hydrogel. Through rational design of functional modules, trivalent iron (Fe^3+^) and 2,3,4-trihydroxybenzaldehyde (THA) form coordination bonds (ophthalmic triol and Fe), and the free amino group of tilapia skin gelatin (Tsg) forms a dynamic Schiff base bond with the carbonyl group of THA. The reversible fracture and reformation of double cross-links not only overcomes the complex process of chemical modification of gelatin, but also realizes the integration of multiple functions on a single platform. In the course of studies, we evaluated the self-healing, injectable, adhesive and mechanical properties of Tsg-THA&Fe hydrogel. The combination of these properties in gelatin-based hydrogels makes them more suitable for the complex wound environment. In terms of biological efficacy evaluation, the biocompatibility, biodegradability, antibacterial and hemostatic properties of Tsg-THA&Fe hydrogel were measured. We further compared the effect of hydrogels with those of commercial hydrocolloid dressings (Comfeel^®^ Plus Transparent), in terms of wound closure, bacterial colonization, and inflammatory response, using a rat back wound model. Furthermore, the toxicity of the Tsg-THA&Fe hydrogel towards major organs was evaluated using in vivo toxicity assays. We aimed to develop a simple and effective method to prepare a Tsg-THA&Fe hydrogel that could serve as an effective biomedical dressing to improve wound healing.

## Experimental section

### Materials

All materials were received from commercial companies without special instructions, and were not purified. 2,3,4-trihydroxybenzaldehyde (THA) (99%), NaCl (99%),CaCl_2_ (99%) and FeCl_3_ (99%) were provided by Shanghai MCLEAN Chemical Company, Shanghai, China, while sodium hydroxide and glacial acetic acid were obtained from Guangzhou XILONG Science Company (Guangzhou, China). 1,1-diphenyl-2picrylhydrazyl (DPPH) was purchased from Shanghai Zhenjun Company (Shanghai, China). Bovine Serum Albumin (BSA) is obtained from Aladdin Biochemical Technology Company (Shanghai, China). Tilapia skin gelatin (Tsg) was prepared as described in our previous study [35]. The Phthalic aldehyde method (OPA) was used to determine the content of free amino in the reaction products [36].

### Preparation of compound THA&Fe

The THA and Fe complexes were explored by referring to experimental methods in the literature [37]. THA was dissolved in deionized water at 70 °C under N_2_ protection, following which anhydrous FeCl_3_ (0.1 M) was applied to the THA solution, at a molar ratio of FeCl_3_ to THA = 1:3. The pH of the mixture was approximately 1, which was not favorable for the oxidation of THA. The mixture was then adjusted to pH 9, by addition of 5 M NaOH and stirred for 3 h to obtain the THA&Fe complexes. The formation of coordination bonds in THA&Fe was determined using Raman spectroscopy [19]. The THA&Fe was lyophilized to obtain THA&Fe powder, which was then pressed into tablets. Raman spectra (LabRAM Aramis spectrometer, Renishaw plc, UK) were acquired on the tablet surface (at least 3 points) with a 785 nm laser line, where the spectrometer pinhole and slit were each set to 400 μm. Each spectrum was irradiated for 500 ms, for an average of 100 times.

### Preparation of hydrogels

The amino group of Tsg was improved according to a previously described method, to achieve the formation of a Schiff base with the carbonyl group of THA [38]. Tsg (5 g) was mixed with deionized water (100 mL) that was heated at 50 °C to obtain a 5% (w v^−1^) solution of Tsg. A certain amount of THA&Fe solution was then mixed into the Tsg solution and stirred for dissolution. The mixed solution was heated at 50℃ for 20 min to prepare the pre-gel. Finally, hydrogel was formed at 25 °C. The corresponding hydrogel is expressed as Tsg-THA&Fex, where x is the amount (μL) of THA&Fe complex added.

### The fourier transform-infrared (FT-IR) spectoscopy test

The lyophilized Tsg and Tsg-THA&Fe40 hydrogel was compressed into KBr trays for spectroscopic measurements in the 4000–400 cm^−1^ range using a FT-IR spectrometer (Nicolet iS50, Thermo Fisher Scientific, USA).

### The scanning electron microscope (SEM)

We prepared Tsg-THA&Fe hydrogel with different compositions. Gold spraying was performed on the cross-sectional surfaces of the lyophilized samples. The mesh structures of the samples were observed using field-emission SEM (S-3000 N, Hitachi, Japan). Image J software (NIH, USA) was employed to measure the pore diameters of hydrogel samples. For each group of samples (at least three samples), three photographs from different areas were documented.

### In vitro swelling test

The wet hydrogels of Tsg-THA&Fe10, Tsg-THA&Fe20, Tsg-THA&Fe30, Tsg-THA&Fe40, and Tsg-THA&Fe50 of the same quality and volume were placed in a bottle containing the simulated wound fluid (SWF; 10 mL; 37 °C) [39]. The SWF consists of an isotonic solution with added protein components. Briefly, 0.4 M NaCl, 0.02 M CaCl_2_ and 2% BSA were mixed and diluted with deionized water. The pH of the original synthetic SWF solution was 7.4 [40]. The hydrogels were removed periodically, and residual water was aspirated from the sample surface. The hydrogels were weighed and the weights were recorded periodically until equilibrium was reached. Each set of tests was repeated five times. The equilibrium swelling rate (ESR) of the hydrogels was calculated using the following equation:

$${\text{ESR}} (\%)= \frac{{W}_{a}-{W}_{b}}{{W}_{b}}\times 100,$$

where W_a_ is the moist weight of the sample after swelling equilibrium and W_b_ is the initial weight of the wet hydrogels.

### In vitro degradation experiments

For in vitro degradation assays, lyophilized Tsg-THA&Fe10, Tsg-THA&Fe20, Tsg-THA&Fe30, Tsg-THA&Fe40 and Tsg-THA&Fe50 hydrogel blocks of the same weight (0.5 g) were placed in 5 mL of PBS (pH 7.4, 37 °C), respectively, and the mixture was shaken uniformly at a rate of 100 r min^−1^ [41]. At scheduled time-points (4, 8, and 12 days), the hydrogel samples were collected, washed with deionized water, freeze-dried, and weighed. The amount of hydrogel degradation was expressed using the following equation:

$$\text{Degradation ratio} \, (\%)=\frac{{W}_{t}}{{W}_{0}}\times 100,$$

where W_0_ and W_t_ are the mass of the original lyophilized hydrogel and the mass of the remaining hydrogel after lyophilization at different degradation times, respectively. All experiments were performed at least five times.

### Test of mechanical and rheological properties

The prepared hydrogels was measured using a rheometer (HAAKE™ MARS™ 40, Thermo Fisher Scientific). Hydrogel were placed between parallel plates (20 mm in diameter) spaced 1 mm apart. Frequency scanning tests were performed at frequencies in the range of 0.1–100 s^−1^ and 1% strain, at 25 °C. To test the mechanical strength of the hydrogels, cylindrical samples (approximately 8 mm height × 10 mm bottom diameter) were prepared for compression measurements. This test was performed using a material testing machine (Instron 3343, Instron, USA), with strains varying from 0 to 90%. All experiments were performed at least three times.

### Hydrogel of injectability and self-healing performance

Tsg-THA&Fe40 hydrogel was injected into a 5 mL syringe at 37 °C on paper with the font "HN920". Meanwhile, Tsg-THA&Fe 40 hydrogel was injected into a vial containing PBS (pH7.4, 37 °C) using a 5 mL syringe, following which observations were made and documented in the form of photographs. Rheological recovery tests were conducted to assess the healing capacity of the hydrogels. The Tsg-THA&Fe40 hydrogel discs were 20 mm in size and 1 mm high. Viscoelastic scanning tests (HAAKE™ MARS™ 40) were performed to determine the viscoelastic areas and fracture strain values, at strains ranging from 0.01 to 1000%. The self-healing behavior was then measured using the Tsg-THA&Fe40, by means of a variable strain scan test (HAAKE™ MARS™ 40) at an angular frequency (1 rad s^−1^). The strain was changed from a low strain (1.0%, 60 s) to a high strain (500%, 60 s), and five cycles were performed [42]. Macroscopic autonomous healing tests were performed to assess the healing capacity of the Tsg-THA&Fe hydrogel. Tsg-THA&Fe40 hydrogel discs with a diameter of 20 mm and height of 5 mm were separated by means of a mid-slit cut, after which they were left to rest for 20 min without external force. Following that, stretching was performed and photographs were taken.

### Hydrogel of adhesion properties

The ability of the Tsg-THA&Fe hydrogel to adhere to the skin tissue was evaluated using clean pig skin [43]. Briefly, the skin tissue was cut into 10 mm × 30 mm rectangles and placed in PBS until use. The Tsg-THA&Fe hydrogel (200 μL) was placed on top of the pig skin, and another piece of pig skin was applied on top of the hydrogel. The bonding region was 10 mm × 10 mm in area. Thereafter, the pig skin was incubated at 37 °C for 3 h. The bonding properties were evaluated on a material testing machine (Instorn 3343), in a splice shear experiment. The measurements were repeated at least three times in all tests. The Tsg-THA&Fe40 hydrogel was made into a cuboidal patch, and the hydrogel was attached to the rat kidney, liver, spleen, and heart. The Tsg-THA&Fe40 hydrogel was attached to the pig skin and human joints. Tissue adhesion capabilities of the hydrogels were observed and photographed.

### Evaluation of in vitro antibacterial effect

*Escherichia coli* (ATCC 25922) and *S. aureus* (ATCC 25923) were used to test the surface antimicrobial effect of the Tsg-THA&Fe hydrogel [44]. Bacterial solutions, such as *E. coli* and *S. aureus* in PBS (pH 7.4), were diluted. Tsg-THA&Fe10, Tsg-THA&Fe20, Tsg-THA&Fe30, Tsg-THA&Fe40 and Tsg-THA&Fe50 hydrogel prepared under sterile conditions were weighed and placed in sterile flasks. A diluted bacterial solution (3 μL) was added to the flasks, while bacterial solution without hydrogel was used as the control group. The flask was placed in a constant-temperature shaking incubator, at 37 °C, and incubated for 24 h. The medium (10 μL) was then removed and diluted with sterilized saline. The diluted solution (10 μL) was uniformly dropped onto agarose plates, at 37 °C, and incubated for 24 h. Each experiment was repeated five times.

### Evaluation of in vitro antioxidant effect

The antioxidant activity of the hydrogels was examined using the method of scavenging stable DPPH radicals [45]. The hydrogels were crushed using a grinder. Appropriate amounts of five different compositions of gelled hydrogels (15 mg) and 100 µM DPPH were dissolved in ethanol (3 mL). The mixture was mixed and reacted in the dark for 0.5 h. The mixture was centrifuged, and the absorbance of the supernatant was measured using a Ultraviolet–visible (UV–Vis) spectrophotometer (Evolution™ 220, Thermo Fisher, USA), at the wavelength of 517 nm. The DPPH scavenging was calculated using the following formula:

$$\text{DPPH scavenging }(\%)=\frac{{A}_{s}-{A}_{c}}{{A}_{s}}\times 100,$$

where A_s_ and A_c_ are the absorbance of the hydrogel (DPPH + ethanol + hydrogel) and vacant (DPPH + ethanol) groups, respectively. Each experiment was repeated three times.

### In vitro cytocompatibility

The cell cytocompatibility of the hydrogels was verified by examining the effect of their extracts on NIH-3T3 mouse fibroblasts cells proliferation [17, 46]. NIH-3T3 mouse fibroblasts cells were kindly provided by Stem Cell Bank, Chinese Academy of Sciences. Freeze-dried Tsg-THA&Fe hydrogel of different compositions were sterilized at 60 °C for 24 h. Thereafter, they were placed in an environment of 37 °C, 5% CO_2_ for 24 h, in Dulbecco’s modified Eagle medium (DMEM), at a concentration of 5 mg mL^−1^, to obtain hydrogel extracts. NIH-3T3 cells (4 × 10^3^ well^−1^) were cultured in DMEM medium containing 10% v/v fetal bovine serum, for 24 h. The original medium was then removed and changed to extract medium, and the blank group was treated with normal medium for 1 and 3 days, respectively. The medium was then replaced with a solution of the 3-(4,5-dimethylthiazole 1-2yl)-2,5-diphenyltetrazolium bromide (MTT; 0.5 mg mL^−1^; 100 μL well^−1^), and incubated for 4 h, at 37 °C with 5% CO_2_, before the solution was removed. The reaction product remaining in each well was dissolved in dimethyl sulfoxide (100 μL) and the well plates were kept in the dark for 15 min. The absorbance of the solution was measured at the wavelength of 570 nm using a microplate reader (Synergy LX, Biotek, USA). The mean ± standard deviation of the absorbance was used to calculate the cytotoxicity in each gel group (*n* = *6*). NIH-3T3 fibroblasts were seeded at a density of 2.0 × 10^4^ cells well^−1^ on hydrogels, for 24 h. After incubation, the cells were stained with a LIVE/DEAD™ Cell Staining Kit (Nanjing Jiancheng, China), to assess cytocompatibility. After incubation with the staining solution for 40 min at 37 °C, the samples were washed thoroughly with PBS and the staining results were photographed using an inverted microscope (IX73, Olympus, Japan).

### In vitro hemocompatibility test

A hemolytic activity assay was performed using rat blood erythrocytes [17]. Rat blood was centrifuged (116×*g*) for 10 min to obtain erythrocytes and washed three times with PBS. The hydrogel (500 µL) was mixed with erythrocyte stock (500 µL) in 2 mL centrifuge tubes and shaken for 1 h, at 37 °C and 100 rpm. Triton™ X-100 (0.1%) was used as a positive control, while PBS buffer was used as a negative control. The mixture was then centrifuged at 1000 rpm for 10 min and the upper supernatant solution (100 µL) was placed in a new 96-well microtiter plate. The absorbance was measured at the wavelength of 540 nm using a microplate reader (Synergy LX, BioTek, USA). The hemolysis rate was calculated using the following equation:

$$\text{Hemolysis rate }(\%)=\frac{{A}_{s}-{A}_{c}}{{A}_{t}-{A}_{c}}\times 100.$$

The absorbance of the sample set was determined as A_s_. A_c_ is the absorbance value of the PBS-treated supernatant, while A_t_ is the absorbance value of the Triton™ X-100 treated supernatant.

### In vivo hemostasis test

Six rats, with three in each group, were anesthetized using 10% chloral hydrate (0.003 v w^−1^), following which 50% of the tail length was cut off with surgical scissors to create a break model of the tail [17]. All the animal experiments in this study were performed in accordance with the guidelines of experimental operations formulated by the Animal Ethics Committee of Hainan University (No: HNUAUCC-2021-00117). After cutting, the wound was covered with the Tsg-THA&Fe40 hydrogel. The change in bleeding during hemostasis was recorded (the bottom was lined with filter paper to aspirate blood, after which the degree of bleeding and hemostasis was quantified before and after the mass change of the filter paper). Untreated wounds were used as the control. The hydrogel’s hemostatic ability was evaluated in hemorrhagic liver rats [17]. Six rats, with three in each group, were anesthetized with a 10% chloral hydrate (0.003 v w^−1^) injection and fixed on a surgical board, following which the rat liver was incised in the abdominal cavity, to remove the perihepatic slurry. The weighed filter paper was then placed in the liver. Liver bleeding was caused using a 5 mm diameter perforator, and a prepared Tsg-THA&Fe40 hydrogel (10 mm diameter) was immediately placed at the bleeding site. The weight of the filter paper from which blood was absorbed until bleeding stopped was measured, and compared with that of the blank group filter paper (in which no treatment was given after needling the liver).

### In vivo biocompatibility test

Ten rats were selected for the in vivo biocompatibility experiments, and the requirements for animal care in trauma experiments were followed [11]. The rats were injected subcutaneously with sterile Tsg-THA&Fe40 hydrogel (200 µL) in dorsal naked skin. Five rats were executed on day 4 and 12 after injection, respectively. Tissues around the implantation site were made and histologically sectioned for hematoxylin–eosin (H&E) staining analysis. Pathological changes were viewed with an inverted microscope (Nikon Eclipse E100, Japan).

### Establishment of rat back wound model

We selected 54 SD pure-line female/male rats, weighing approximately 300 g. The rats were kept at room temperature (23 °C) for 5 days, for acclimatization, and then divided into three groups, with 18 rats in each group. The wound models were created after 2 days. The rats were anesthetized with 10% (w/v) chloral hydrate. Part of the hair in the back area was then quickly removed, following which the surgical area at the same position on the right side of the back was disinfected with 75% alcohol, and full-thickness round skin resection with a diameter of approximately 1.5 cm was performed. On day 0 after surgery, the same dosage of different hydrogels was used for wound treatment for 12 days, to explore the effect of the hydrogels on skin tissue healing [47].

### Observation of wound healing and calculation of healing rate

After surgery, the wounds of different groups were covered with Tsg-THA&Fe40 hydrogel (group E) and positive control Comfeel^®^ Plus Transparent (group P) treatments; the blank group (group C) was without any treatment. Each sample was separated to prevent contact. The trauma surface was photographed on days 0, 4, 8, and 12 after surgery, and the trauma area of each group was measured by means of digital photography, using Image J software (NIH, USA).

$$\text{Wound contraction rate }(\%)=\frac{{S}_{0}-{S}_{n}}{{S}_{0}}\times 100,$$

where S_0_ is the trauma area on day 0 and S_n_ is the trauma area on day n. The wound contraction rate has been represented as mean ± standard deviation (*n* = *6*).

### Analysis of wound histology and immunology

The wound tissues of the group C, group P, and group E groups were collected in a rat wound model experiment, and wound histology and immunoassays were performed. The wound tissues of each group were fixed with 4% paraformaldehyde and sectioned by means of paraffin embedding. Sections were stained with H&E and Masson’s trichrome. Finally, a microscope (Eclipse E100, Nikon) was used for detection and an image acquisition system (DS-U3, Nikon) was used for analysis. Immunofluorescence analysis was performed on wound tissues, which mainly include CD206, CD68, CD31, and alpha smooth muscle actin (α-SMA). Paraffin sections embedded in skin tissue were successively placed in xylene, anhydrous ethanol, and 75% alcohol solution, following which they were dewaxed in water. Tissue sections were placed in a repair box with antigen repair solution (EDTA, pH 8.0), for antigen repair. The slices were dried and then circled with a histochemical pen, and Bull Serum Albumin (BSA) was closed for 30 min. The tissue sections were then incubated with anti-CD68 rabbit polyclonal antibody (pAb), anti-CD206 rabbit pAb, anti-CD31 rabbit pAb, and anti-α-SMA rabbit pAb mixed in proportion with PBS, overnight at 4 °C in a wet box, following which they were covered with rabbit pAb (primary antibody of the corresponding species) and incubated in the dark for 50 min. 4′,6-diamidino-2-phenylindole (DAPI) was used to re-dye the nuclei and quench the tissue for self-fluorescence, before sealing. Finally, the collected images were observed using a fluorescence microscope (Eclipse E100, Nikon). There were four samples in each group, three images were collected from each sample, and the number of co-expressed cells in the CD68 and CD206 images was counted using Image J. At the same time, the relative fluorescence intensities of the CD31 and α-SMA images were quantified.

The protein concentrations of interleukin-6 (IL-6), interleukin-10 (IL-10), interleukin-1β (IL-1β), tumor necrosis factor-alpha (TNF-α), arginase-1 (Arg-1), tumor growth factor-beta (TGF-β), vascular endothelial growth factor (VEGF), signal transducer and activators of transcription 6 (STAT6), lipopolysaccharide (LPS), toll-like receptor 2 (TLR2) and toll-like receptor 4 (TLR4) in the wound tissues were measured using an enzyme-linked immunoassay (ELISA) kit (Shanghai X-Y Biotechnology, China). First, tissue samples from the skin wounds of each group were weighed and added to PBS (pH 7.4) for homogenization, followed by centrifugation for 20 min to collect the supernatant. Using an ELISA kit, the supernatant was diluted, added to the plate, and incubated at 37 °C for 30 min. The plate was then washed using a washing solution, following which an enzyme standard reagent was added to it, and incubated at 37 °C for 30 min. Next, the plate was washed again, color solution was added to it, and the reaction was carried out at 37 °C for 15 min. Finally, stop solution was added to stop the reaction. The absorbance was measured at the wavelength of 450 nm using a microplate reader. Simultaneously, we determined the standard curve of the protein under the same conditions, using a standard substance. To better evaluate the content of immune factor proteins in the wound tissue, we measured the total protein content in wound tissues of the same quality. The protein levels of IL-6, IL-10, IL-1β, TNF-α, IL-10, Arg-1, TGF-β, STAT6, VEGF, LPS, TLR2, and TLR4 in the wound tissue were calculated.

### High-throughput sequencing and bioinformatic analysis from wound microorganisms

Microbial changes in each group of wounds were studied using gene sequencing technology, on each group of skin tissues sampled. To facilitate the comparison and analysis of the data in each group, the labels of each group were defined as XY.n, by means of bioinformatic analysis. The X-position mainly represents the untreated (group C), Comfeel^®^ Plus Transparent treatment (group P), and Tsg-THA&Fe 40 hydrogel treatment (group E) groups. The Y-positions represent different time-points, including days 4, 8, and 12 days. The n-position represents the serial number of the different samples in the group. First, genomic DNA was extracted from each sample and the PCR products were purified by means of electrophoretic detection. A gene-sequencing library was generated using a sample preparation kit. After the quality of the gene library was assessed using a bioanalytical instrument, the NovaSeq (Nuohe, Beijing, China) platform was used to sequence the library and obtain end sequences that could be paired. Next, the terminal sequences were truncated by the primer sequences, and the same gene fragments were read using paired-ends, to form the original tags of the sample genome [48]. The Quantitative Insights Into Microbial Ecology (QIIME) system (version 1.9.1) filtered the original tags under specific settings, whereas the UCHIME algorithm model generated effective tags by comparing them with the original database and eliminating the chimeric sequences [49–52]. Finally, sequences with > 97% similarity were categorized using Uparse software, to form OperationalTaxonomic Units (OTUs) [53]. Representative gene sequences were annotated with taxonomic information using the Mothur algorithm database [54].

To analyze species differences between samples, we categorized and integrated different OTUs using the MUSCLE software (Version 3.8.31), to facilitate differentiation among microbial species [55]. Based on the integration of the genomic data of each previous sample, we analyzed the genomic data of the samples using bioinformatics methods. Alpha diversity was used to analyze the diversity of species, in which the complexity of the microbial diversity of the samples was analyzed using indicators such as the Shannon index. Each dataset was calculated using QIIME (version 1.9.1) and displayed using R software (version 2.15.3). Beta diversity was analyzed for structural differences in the microbial communities of the samples, and QIIME software (version 1.9.1) was used to analyze the differences between weighted and unweighted Unifrac on the samples. Multivariate statistical analyses were performed to quantify the variability between the groups. Non-metric multidimensional scaling (NMDS) analysis was performed by displaying sample data as points on a multidimensional spatial scale, with the distance between points indicating the differences between samples, as visualized using R software (version 2.15.3) [56]. We also performed an unweighted pair-group method with arithmetic means (UPGMA) analysis to show the differentiation of the main species among the groups. Correlation analysis is a statistical method used to determine the correlation between a sample microorganism and environmental factors. A canonical correspondence analysis (CCA) ordination diagram is a double-order diagram that plots species, samples, and environmental factors on a single graph, to view the relationships between species distribution, community distribution, and environmental factors, where CCA1 indicates the correlation of sample microorganisms and environmental factors with the first ordination axis and CCA2 indicates the correlation with the second ordination axis [57]. In addition, statistical analysis with the envfit function demonstrated the degree of influence of environmental factors on sample microorganisms.

### In vivo safety assessment of hydrogels

Twelve rats were selected to assess the in vivo toxicity of the hydrogel in a rat full-laye**r** trauma model. They were randomly divided into normal (group N), untreated wound (group C), and Tsg-THA&Fe40-treated (group E) groups. On day 12, 2 mL of animal blood was collected from each group, for biochemical and hematological tests. The animals were subsequently sacrificed, and sections of their liver, kidney, and spleen tissues were stained with H&E, to monitor the histomorphological changes. Among the blood biochemical and hematological tests, the main indicators included, white blood cells (WBC), neutrophils (Neut), platelets (PLT), lymphocytes (Lymph), monocytes (Mon), red blood cell count (RBC), hemoglobin (HGB), red blood cell pressure product (HCT), platelets (PLT), blood urea nitrogen (BUN), creatinine (CREA), glutamate transaminase (ALT), glutamic oxalacetic transaminase (AST) and other biochemical indicators, were measured.

### Data analysis

The analyzed data are expressed as at least three replicate sample means ± standard deviations. One-way ANOVA was performed for each group of data using SPSS (Windows, IBM, USA), where asterisks indicate significant differences (*P < 0.05, **P < 0.01, and ***P < 0.001).

## Results and discussion

### Preparation of the physicochemical double-cross-linked gelatin-based hydrogels

**Fig. 1:**
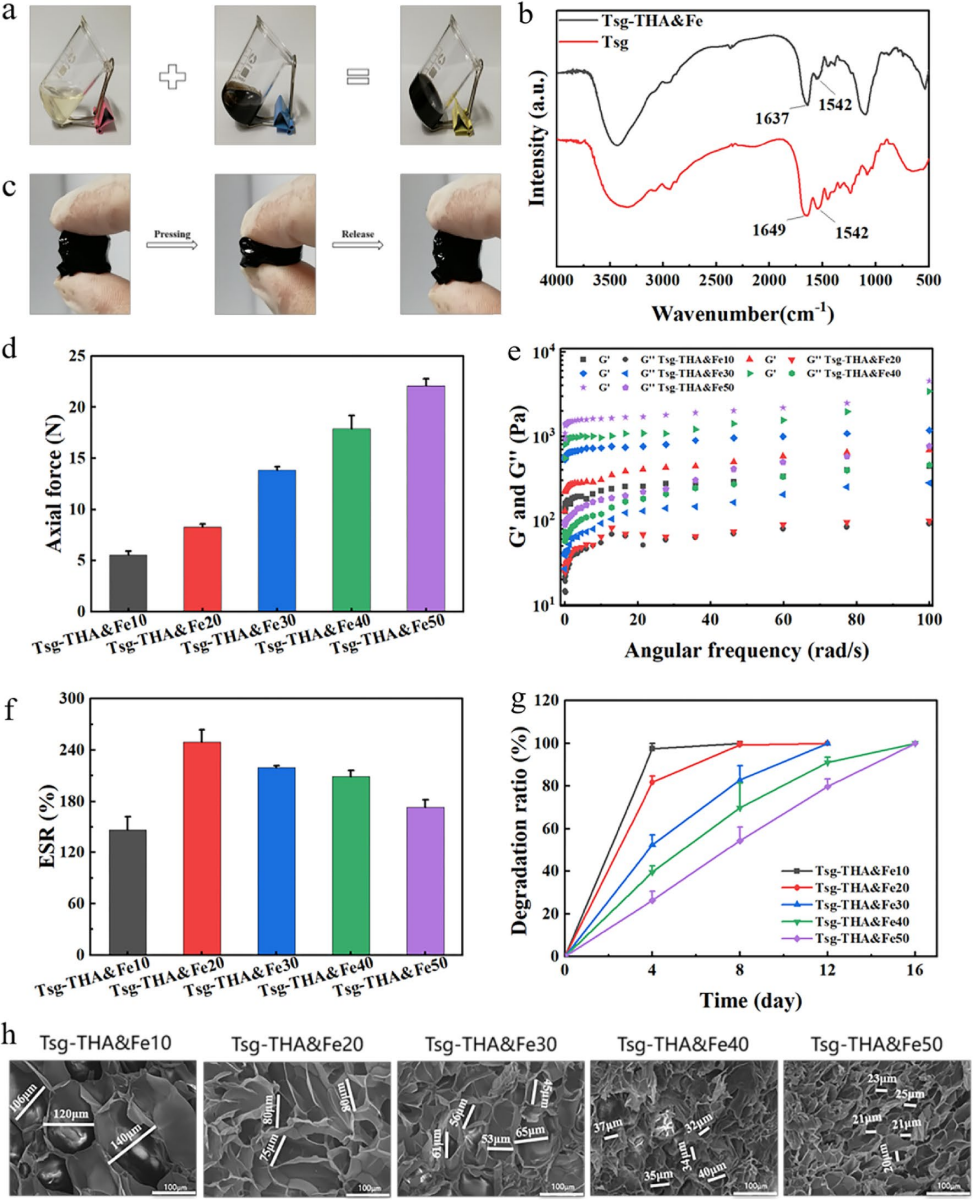
Preparation and characterization of the Tsg-THA&Fe hydrogel. **a** Photographs of Tsg solution, THA&Fe solution and the formed Tsg-THA&Fe hydrogel. **b** FT-IR spectra of Tsg and Tsg-THA&Fe hydrogel. **c** The original compressed and recovered states of the prepared hydrogels. **d** The compression force of the Tsg-THA&Fe hydrogel at 90% strain (*n* = *3*). **e** Frequency dependence of the dynamic storage modulus (G′) and loss modulus (G″) of Tsg-THA&Fe hydrogel. **f** The equilibrium swelling rate (ESR) of Tsg-THA&Fe hydrogels (*n* = *5*). **g** In vitro degradation properties of Tsg-THA&Fe hydrogel with pH 7.4 at 37 °C (*n* = *5*). **h** The SEM images of Tsg-THA&Fe hydrogel

A double-cross-linked gelatin-based multifunctional hydrogel with good biocompatibility, antibacterial properties, and adhesion performance was prepared from the complex of fish skin gelatin (Tsg) and stable THA&Fe, according to the characteristics of skin wounds (Fig. 1a). The Tsg was prepared from tilapia skin after extraction, as described in our previous work, and confirmed using the phthalic aldehyde method (OPA) on Tsgfree amino content, which was 4 mmol g^−1^ in Tsg [35, 36]. In an aqueous solution with pH 9, THA formed stable ternary complex molecules with Fe^3+^, at a molar ratio of 3:1. The formation of THA and Fe coordination bonds was confirmed by the presence of characteristic peaks at 500–680 and 1200–1500 cm^–1^, upon analysis using Raman spectroscopy (Additional file 1: Fig. S1a) [58, 59]. Tsg and THA&Fe were then mixed by means of Schiff base bonding, to produce the double-cross-linked gelatin-based hydrogels (Fig. 1a); different groups of Tsg-THA&Fex (where x is the amount (μL) of THA&Fe complex added) hydrogels can be prepared using different dosage of THA&Fe (Additional file 1: Table S1). The simple double-cross-linked structure of Schiff base and coordination bond enhances not only the physical properties, but also the biological efficacy of the hydrogels. The cross-linking of Schiff base bonds is slow, whereas gelation is accelerated by the addition of coordination bonds. The introduction of coordination bonds provides robust adhesion and antibacterial effects. Moreover, the addition of THA and Fe coordination bonds, which avoid the carbon–carbon double bonds used in most hydrogels, also improves the mechanical properties of the hydrogel network [60, 61]. The Fourier transform-infrared (FT-IR) spectrum in Fig. 1b shows that the characteristic peaks of Tsg at 1649 and 1542 cm^−1^ represent the tensile vibration of C=O in amide I and the bending vibration of N–H in amide II, respectively [62, 63]. In comparison to the characteristic peak in Tsg, it was found that the peak of the spectrum of Tsg-THA&Fe hydrogel was significantly weakened at 1542 cm^−1^, which proved that the N–H in Tsg-THA&Fe hydrogel was cross-linked [64]. At the same time, the peak intensity at 1637 cm^−1^ in the spectrograph of Tsg-THA&Fe hydrogel was significantly higher than that of amide I in Tsg, indicating the formation of C=N in Tsg-THA&Fe hydrogel [7, 24]. The double-cross-linked Tsg-THA&Fe40 hydrogel was chosen as a model sample that could withstand finger-to-finger adhesion and compression (Fig. 1c). The cylindrical hydrogel (10 mm diameter) was compressed at 0–90% strain in the material test system (Fig. 1d and Additional file 1: Fig. S1b). As the addition of THA&Fe increased from 10 to 50 μL, the maximum compressive stress of Tsg-THA&Fe hydrogel increased from 5 to 23 N, indicating an increase in the cross-linking density of the hydrogel. The mechanical properties of Tsg-THA&Fe hydrogel are better than those of modified chitosan hydrogel (3–15 N) [17].

### Hydrogels formation time, rheological properties, morphology, swelling, and in vitro degradation behavior of the hydrogels

The controllability and appropriateness of the gelation time of hydrogels are crucial for their application as biomedical materials. The gelation time of the hydrogels decreased from 30 to 5 min with increasing THA&Fe content (Additional file 1: Table S1). The accelerated gelation rate may be related to the increase in the number of Schiff base bonds and formation of coordination bonds. To assess the viscoelasticity of the hydrogels, the storage modulus (G′) and loss modulus (G″) of the hydrogels were examined using rheometry with frequency (Fig. 1e). The results found that upon a frequency scan from 0.1 to 100 rad s^−1^, the G′ value increased from 139 to 1029 Pa, with the G′ value always greater than the G″ value in different groups of Tsg-THA&Fe hydrogel, indicating that Tsg-THA&Fe hydrogel have typical viscoelastic characteristics. Hydrogel, as a wound dressing, can use its three-dimensional network structure to absorb tissue exudate from the wound, keep the environment moist, and improve the wound healing environment. The swelling rate of the Tsg-THA&Fe hydrogel was tested in SWF at 37 °C, in an equilibrium state. After the Tsg-THA&Fe hydrogel were immersed in SWF for 24 h, the wet hydrogels of Tsg-THA&Fe20,Tsg-THA&Fe30,Tsg-THA&Fe40 and Tsg-THA&Fe50 showed a decrease in ESR from 249 to 173%, which may be related to the increased cross-link density of the hydrogels. The Tsg-THA&Fe10 with the lowest crosslink density showed a lower ESR (146%) than the other hydrogel samples, which may be due to the fact that the loose network structure is more easily disrupted. (Fig. 1f). The degradability of wound-dressing-based hydrogels is an essential feature in biomedical applications, to enhance their flexibility and increase their range of utilization. Upon evaluation of the degradation behavior of the resulting Tsg-THA&Fe hydrogel, the degradation time of the Tsg-THA&Fe hydrogel was found to increase with increasing cross-linking density (Fig. 1g). Tsg-THA&Fe10 and Tsg-THA&Fe20 hydrogel decomposed entirely in less than a week, while the other three hydrogels had a slower degradation time, with complete degradation taking about 8–16 days, thereby showing promise for in vivo wound healing applications in traumatic wounds. The internal morphological structure of the Tsg-THA&Fe hydrogel was demonstrated using scanning electron microscopy (SEM). The results showed that the Tsg-THA&Fe hydrogel had a three-dimensional mesh structure (Fig. 1h and Additional file 1: Fig. S2a), and the pore size of the Tsg-THA&Fe hydrogel decreased gradually with increasing THA&Fe content. The change in pore size may be related to the increase in the cross-linking density in the network, which is consistent with the results of the changes in the storage modulus and swelling rate.

### Injectability and self-healing properties of the hydrogels

**Fig. 2:**
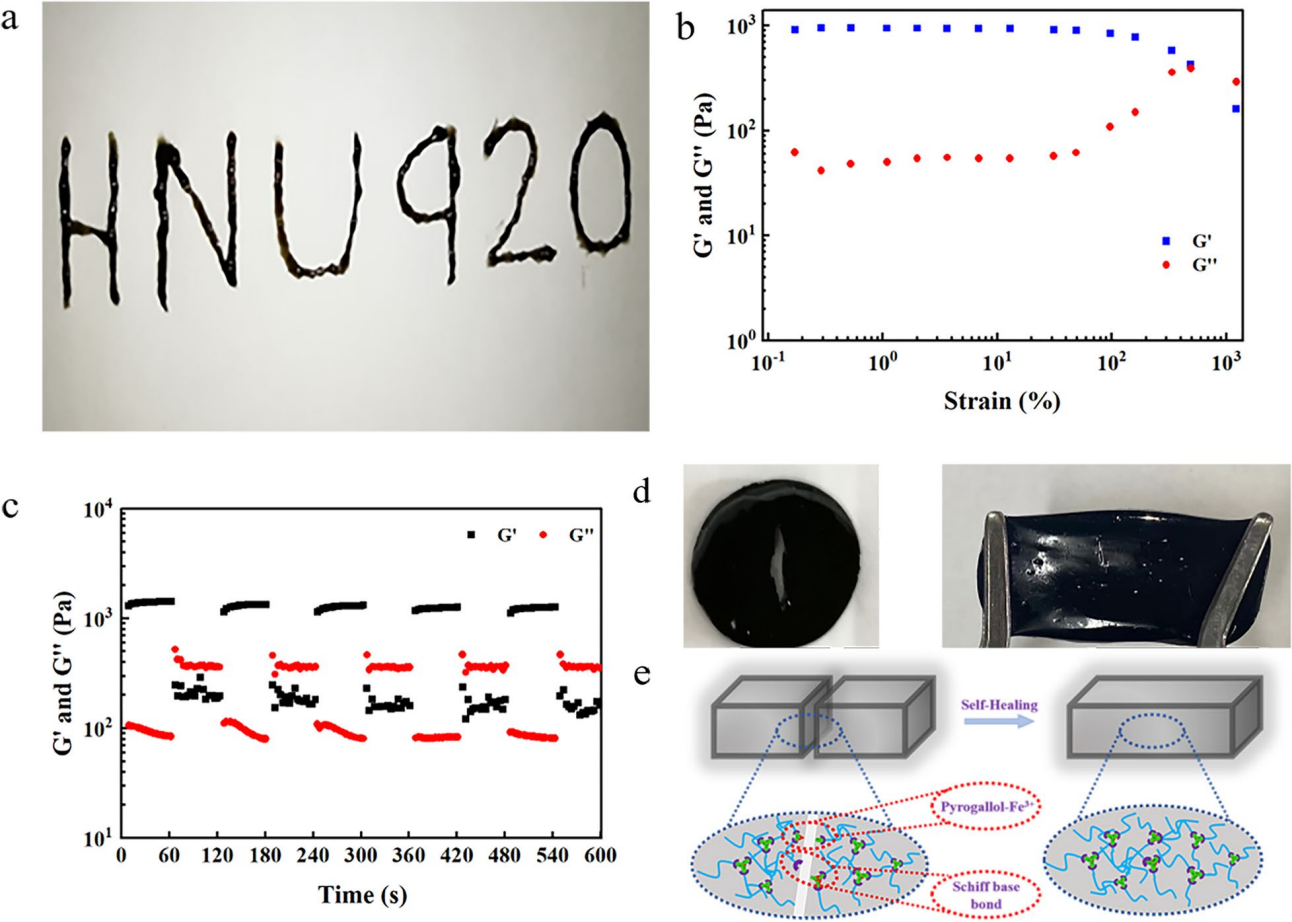
Injectable and self-healing properties of the hydrogels. **a** Injectable properties of Tsg-THA&Fe hydrogel under normal environment. **b** Strain scan of Tsg-THA&Fe40 hydrogel at G′ and G″. **c** Rheological properties of the Tsg-THA&Fe40 hydrogel at alternating strain switching from 1 to 500%. **d** Demonstration of self-healing of hydrogels with cracks. e) Self-healing mechanism of Tsg-THA&Fe hydrogel

Conventional hydrogels break under external forces and cannot completely self-repair. When the Tsg-THA&Fe40 hydrogel was injected continuously using a syringe into PBS (37 °C, pH 7.4) and the word "HNU920" was drawn, the hydrogel remained in a stable gel state after the shear force was removed (Fig. 2a and Additional file 1: Fig. S2b). The Tsg-THA&Fe hydrogel have good shape adaptation and autonomous healing properties, which allows them to be self-healing, prolong the service life, and improve the wound healing conditions.

To evaluate the self-healing performance of the Tsg-THA&Fe hydrogel, we selected Tsg-THA&Fe40 hydrogel as a representative for rheological recovery tests and macroscopic self-healing experiments. At strains below 1000%, the strain scan of the Tsg-THA&Fe40 hydrogel showed the intersection of G′ and G″ (Fig. 2b). This is critical for hydrogel network disruption. The sudden decrease in the G' and G'' values at strain indicates the collapse of the Tsg-THA&Fe40 hydrogel structural network and the transformation from a solid with gel-like properties to a liquid with fluid-like properties [7, 65]. Continuous strain recovery scanning experiments were then performed to determine the autonomous healing behavior of the Tsg-THA&Fe hydrogel, from a rheological perspective (Fig. 2c). When high strain (500%) was applied, the G′ value decreased from 1000 to 200 Pa below G″, indicating that the network structure of the Tsg-THA&Fe40 hydrogel was disrupted. When the 500% strain was restored to 1%, G′ and G″ still returned to their original values even after five cycles of 60 s alternate intervals, which indicated that the Tsg-THA&Fe40 hydrogel structure was reconstructed. Repeated damage healing experiments formally demonstrated the good self-healing properties of the Tsg-THA&Fe hydrogel. Macroscopic self-healing experiments were performed to evaluate the self-healing performance of the hydrogels (Fig. 2d). The hydrogel sheet Tsg-THA&Fe40 hydrogel with cracks could fully recover and re-form the hydrogel discs, after 20 min of standing at room temperature, and in addition, could be stretched without cracks forming at the hydrogel bond.

These results showed that the Tsg-THA&Fe hydrogel have good injectability and self-healing properties. The hydrogel forms a dynamic Schiff base bond between the amino group of Tsg and the aldehyde group of THA, and a dynamic coordination bond between the o-phenyl triol of THA and Fe^3+^. The dynamic equilibrium ability of the two dynamic bonds increases the rapid network recovery of the hydrogel (Fig. 2e) [7]. Thus, Tsg-THA&Fe hydrogel can adapt to complex wound environments and have potential for application as wound dressings.

### Hemocompatibility, cytocompatibility, and in vivo compatibility of hydrogels

**Fig. 3:**
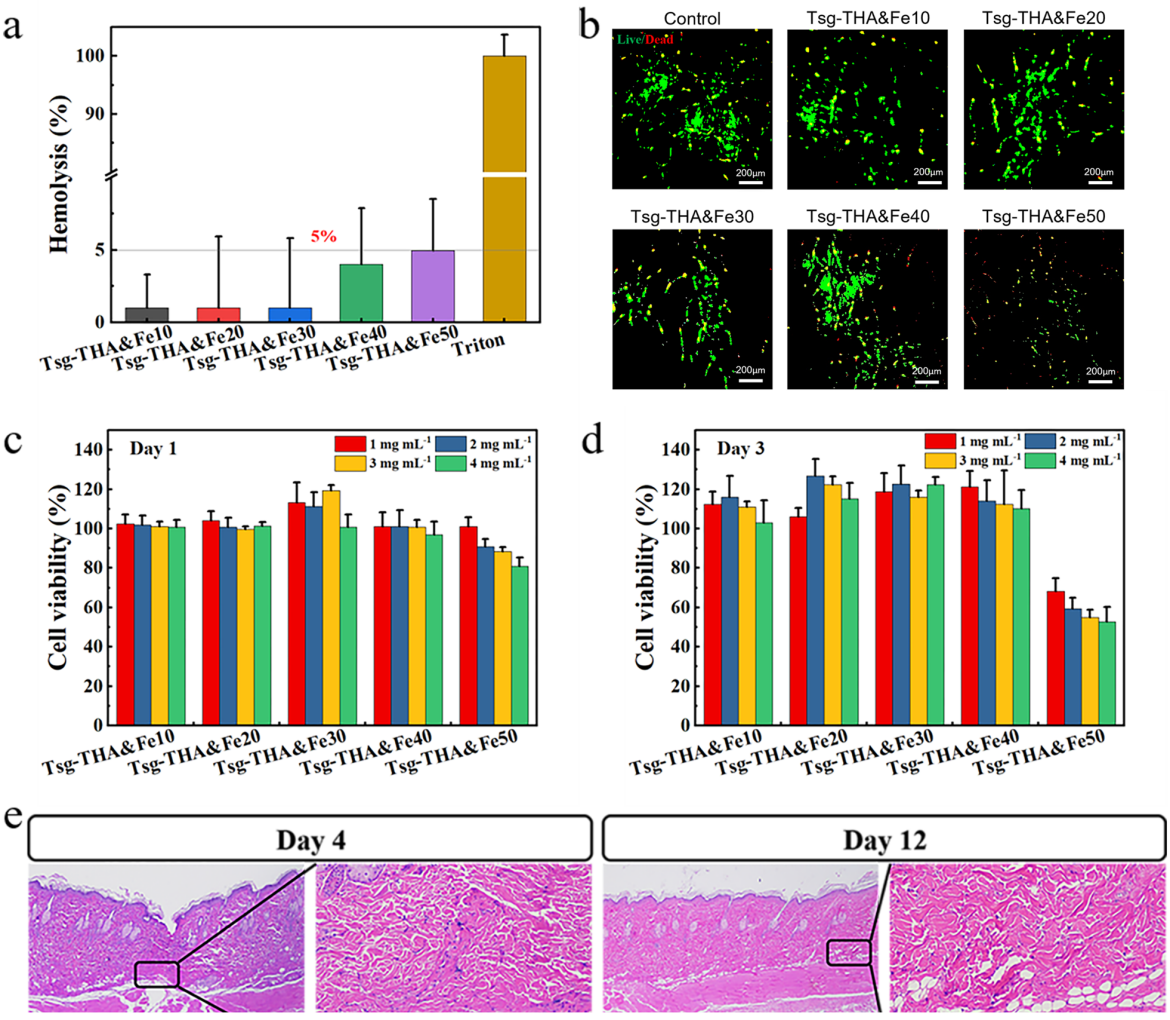
Biocompatibility of the Tsg-THA&Fe hydrogel. **a** Hemolysis assay of Tsg-THA&Fe hydrogel (*n* = *3*). **b** Cell staining of NIH-3T3 cells cultured in the Tsg-THA&Fe hydrogel for 3 days. Survival rate of NIH-3T3 cells cultured in each group at different concentrations of hydrogel leachate for 1 **c** and 3 **d** days (*n* = *6*). **e** Hematoxylin–eosin (H&E) staining of skin tissue implanted subcutaneously with Tsg-THA&Fe40 hydrogel, the box shows the approximate location of hydrogel implantation (*n* = *5*)

Good biocompatibility is essential for biomedical hydrogel wound dressing. As shown in Fig. 3, with an increase in the THA&Fe content, there were no significant changes in these groups (Additional file 1: Fig. S3), and the hemolysis rate was below 5%, indicating that Tsg-THA&Fe hydrogel have good hemocompatibility (Fig. 3a) [66].

The biocompatibility of a material is also an important criterion for assessing its feasibility for biomedical applications. Cell morphology and viability were analyzed by LIVE/DEAD™ cell staining [67]. Therefore, the survival status of NIH-3T3 cells incubated with Tsg-THA&Fe hydrogel for 72 h was observed by LIVE/DEAD™ cell staining. Meanwhile, the 3-(4,5-dimethylthiazole 1-2yl)-2,5-diphenyltetrazolium bromide (MTT) assay was used to evaluate the metabolic activity of NIH-3T3 cells after 1 and 3 days of treatment with Tsg-THA&Fe hydrogel extract at different concentrations [67]. The results showed that almost all NIH-3T3 cells were stained green (live cells), while only a few cells were stained red (dead cells), except in the Tsg-THA&Fe50 hydrogel group (Fig. 3b). In addition, MTT test results showed that (Fig. 3c), compared to the blank group on days 1 and 3, the Tsg-THA&Fe10, Tsg-THA&Fe20, Tsg-THA&Fe30, and Tsg-THA&Fe40 hydrogel groups did not significantly inhibit the cell viability of NIH-3T3 cells cultured at different concentrations for 1 and 3 days. Notably, on day 1 of culture, Tsg-THA&Fe50 hydrogel showed inhibition of NIH-3T3 cell metabolic activity with increasing concentrations, as compared to the activity in the blank group on day 1. On day 3, the cell viability of NIH-3T3 cells was markedly inhibited in the Tsg-THA&Fe50 hydrogel group, as compared with that in the blank group, on day 3. These results indicated that the Tsg-THA&Fe hydrogel had good cytocompatibility, with a certain amount of THA&Fe.

Hematoxylin–eosin (H&E) staining was used to observe the histomorphological changes on day 12 after subcutaneous injection of the Tsg-THA&Fe40 hydrogel (Fig. 3e). After 12 days, no obvious inflammatory cell infiltration, cell degeneration, or tissue necrosis were found in the skin tissue, indicating that the Tsg-THA&Fe hydrogel has good compatibility in vivo.

### In vitro antioxidant and antibacterial properties of hydrogels

**Fig. 4:**
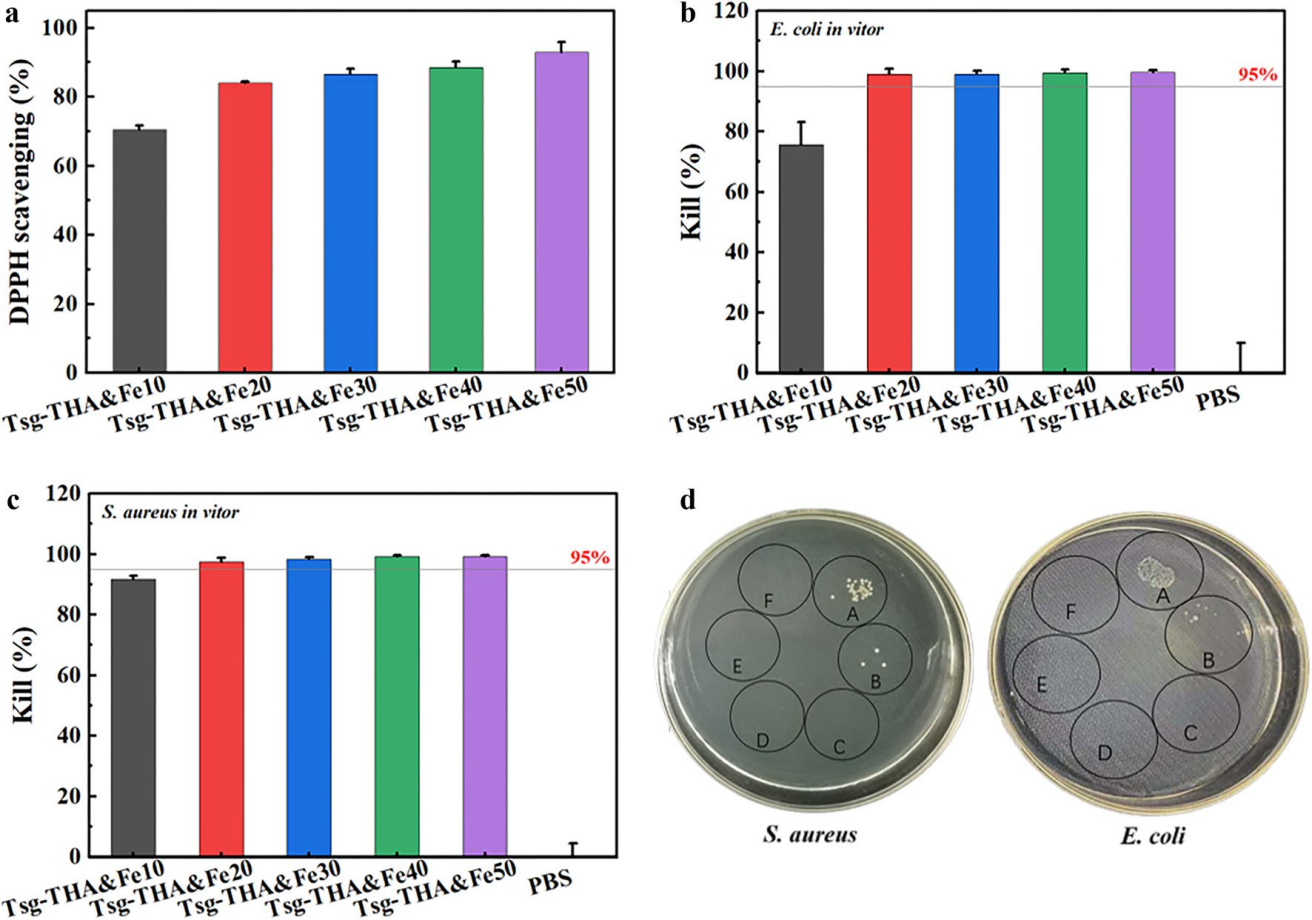
In vitro antioxidant and bacterial inhibition properties of the hydrogels. **a** DPPH scavenging of Tsg-THA&Fe hydrogel (*n* = *3*). Quantitative results of the inhibition performance of Tsg-THA&Fe hydrogel against **b** *E. coli* and **c** *S. aureus* (*n* = *5*). **d** Images of bacterial clones formed by the inhibitory effects of the control group (**A**), Tsg-THA&Fe10 hydrogel group (**B**), Tsg-THA&Fe20 hydrogel group (**C**), Tsg-THA&Fe30 hydrogel group (**D**), Tsg-THA&Fe40 hydrogel group (**E**), Tsg-THA&Fe50 hydrogel group (**F**) on *S. aureus* and *E. coli* on agar plates are shown

Excessive accumulation of free radicals generated at the wound site triggers oxidative stress, and causes cytotoxicity by damaging the enzymes and DNA [68]. Wound dressings can promote healing by scavenging excess free radicals, such as reactive oxygen species (ROS) [69]. The antioxidant properties of the hydrogels were determined by assessing the free radical 1,1-diphenyl-2picrylhydrazyl (DPPH) scavenging effect. All groups of hydrogels showed good free radical-scavenging ability (> 80%), and a significant increase in the free radical-scavenging ability of the hydrogels occurred with increasing THA&Fe content (Fig. 4a). In addition to the free radical-scavenging ability of gelatin in hydrogels, THA&Fe was also effective in scavenging DPPH [70]. Thus, the interplay of Tsg and THA&Fe equipped the hydrogel with better antioxidant activity, which is very useful for wound healing applications [27].

During wound treatment, traumatic wounds exude tissue fluid, which increases the risk of pathogenic bacterial infections, and remains one of the main obstacles to wound healing [71]. The inherent antimicrobial capacity of hydrogel dressings is essential to prevent the invasion of external bacteria and their adhesion to the wound. The in vitro antimicrobial activity of hydrogels was tested against *E. coli* and *S. aureus*. Upon reaction for 24 h at 37 °C, Tsg-THA&Fe10 hydrogel killed 75% of *E. coli* and 70% of *S. aureus*, while the remaining groups of *E. coli* (> 95%) and *S. aureus* (> 95%) were inactivated (Fig. 4b, c). These results indicated that the Tsg-THA&Fe hydrogel exhibited excellent in vitro antibacterial performance against *E. coli* and *S. aureus*. As compared to the blank group, almost no bacterial colonization was observed on the agar plates of the hydrogel-treated group (Fig. 4d). In addition, the antimicrobial effect improved with an increase in THA&Fe content. The potent antimicrobial properties may be attributed to the Schiff base compounds generated by the reaction of Tsg with THA&Fe. Fe^3+^ is a non-toxic antimicrobial agent that can react with amino and sulfhydryl groups on intracellular proteins and bind to the active center of the protein, thereby disrupting the normal physiological structure of bacteria and leading to microbial death [72–74]. The synergistic effect of the Schiff base compounds with Fe^3+^ enhanced the antibacterial effect. Thus, Tsg-THA&Fe hydrogel showed great potential in preventing the invasion of external microorganisms and avoiding infections.

### Adhesive properties and hemostatic ability of hydrogels

**Fig. 5:**
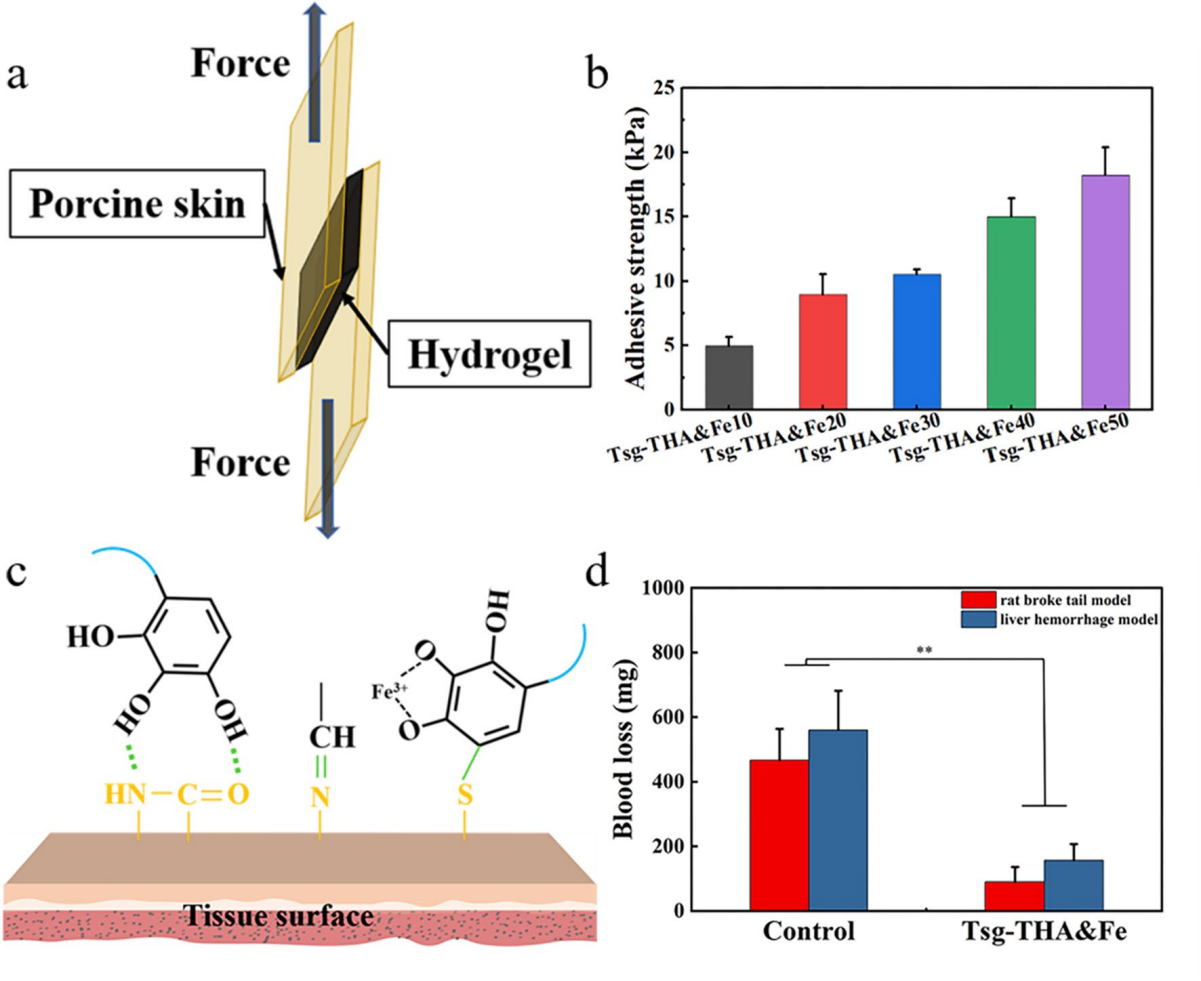
Adhesive and hemostatic properties of the Tsg-THA&Fe. **a** Schematic diagram of the lap shear test of Tsg-THA&Fe hydrogel by using pigskin. **b** Adhesion strength test of Tsg-THA&Fe to pigskin *(n* = *3)*. **c** Skin adhesion mechanism of Tsg-THA&Fe. **d** The hemostatic effect was evaluated in the rat broke tail model and liver hemorrhage model (*n* = 3, *P < 0.05, **P < *0.01*)

Wounds are a dynamic environment. Conventional wound dressings form an unstable connection to the wound surface, thereby slowing the healing process. A dressing with tissue adhesion bonds can ensure a good healing environment, which in turn prevents wound infection and improves wound healing. The adhesion properties of the Tsg-THA&Fe hydrogel were evaluated using a lap-shear assay (Fig. 5a). The adhesive strength of the Tsg-THA&Fe hydrogel increased from 4.97 to 18.23 kPa with increasing THA&Fe content (Fig. 5b). The bonding strength of Tsg-THA&Fe50 hydrogel obtained by lap shear test was close to that of gold standard fibrin adhesive (15.4 ± 2.8 kPa) [75–77]. The adhesive strength of a hydrogel depends on its cross-linking density and interfacial adhesion strength. The increase in the cross-linking density enhanced the mechanical strength of the hydrogel, while the adhesion of aldehyde and phenolic hydroxyl groups increased the interfacial adhesion strength of the hydrogel (Fig. 5c). When the adhesion ability of the Tsg-THA&Fe hydrogel was further evaluated in terms of tissue adhesion, the Tsg-THA&Fe40 hydrogel were found to firmly adhere to freely curved finger joints and twisted pig skin planes (Additional file 1: Fig. S4a, b). The Tsg-THA&Fe hydrogel showed excellent adhesion to various tissues, without an external support (Additional file 1: Fig. S4c). These results suggested that the Tsg-THA&Fe hydrogel with high adhesive strength have good potential for application in dynamic wound dressings.

In studies, gelatin with good biocompatibility has been found to have hemostatic properties. Gelatin-based hydrogels with excellent adhesion properties have been prepared to act as a physical barrier and provide hemostasis [78, 79]. The hemostatic properties of the Tsg-THA&Fe hydrogel were evaluated by recording the blood loss during the treatment, using a rat tail break experiment and a liver hemorrhage model (Additional file 1: Fig. S5). In the tail break model, bleeding was significantly reduced in the Tsg-THA&Fe hydrogel group (by 404 mg; *P* < *0.05*), whereas significant bleeding occurred in the untreated group. In the hepatic hemorrhage model, better hemostasis was observed in the hydrogel-treated group, with a significant reduction in blood loss (of 376 mg; *P* < *0.05*) (Fig. 5d). Erythrocyte aggregation and platelet adhesion induced by the amino group, as well as the excellent adhesive properties of the Tsg-THA&Fe hydrogel, were combined to improve the hemostatic performance of the Tsg-THA&Fe hydrogel [80].

### In vivo wound healing

**Fig. 6:**
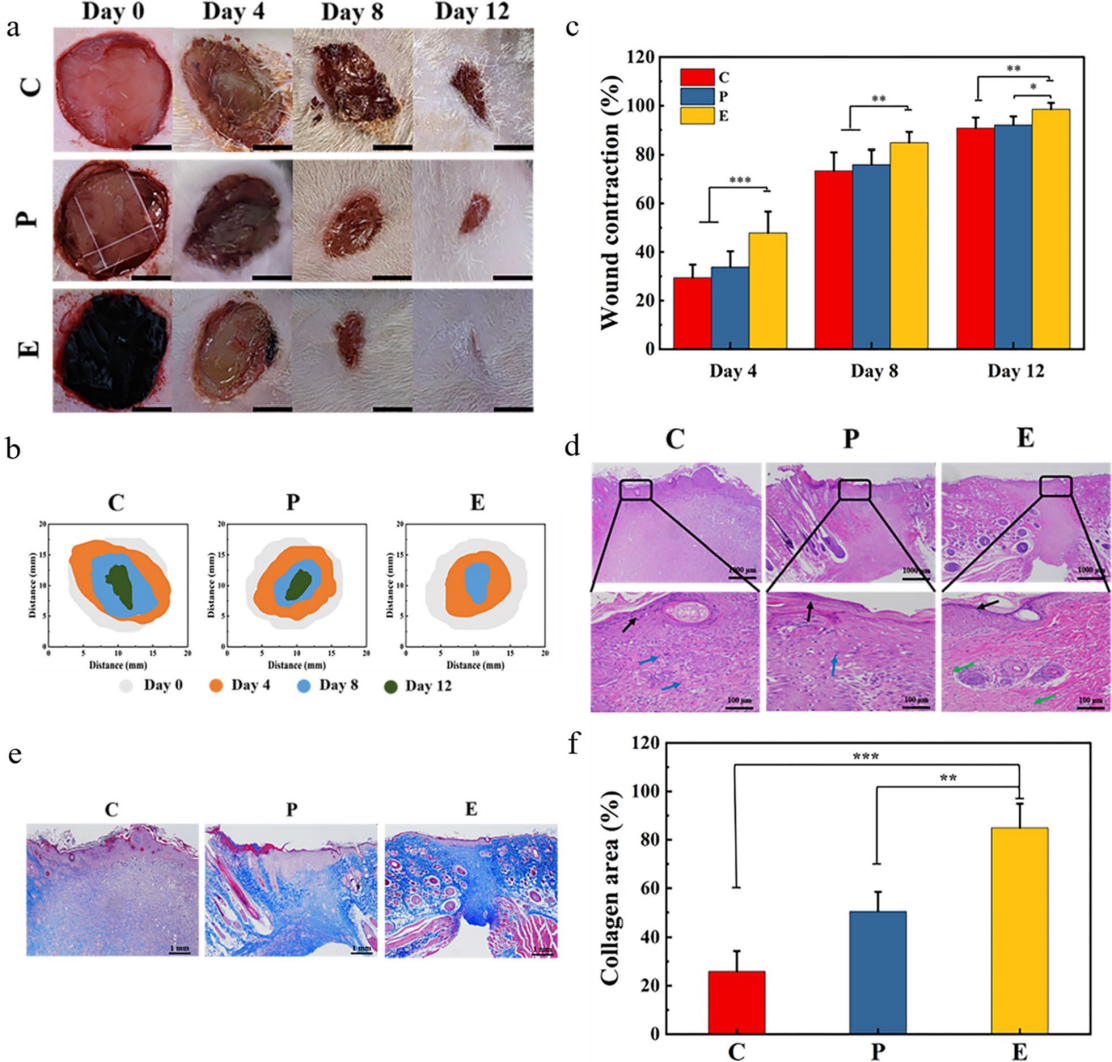
Evaluation of the effect of the Tsg-THA&Fe hydrogel on wound healing in vivo. **a** Wounds of untreated wound group (group C), Comfeel^®^ Plus Transparent treatment group (group P) and Tsg-THA&Fe40 hydrogel treatment group (group E) on day 4, 8 and 12. (scale bar: 5 mm). **b** Schematic diagrams of contraction changes in the groups C, P, and E on days 4, 8, and 12 during wound healing. **c** Wound contraction rates of each group at days 4, 8, and 12 (*n* = *6*). **d** H&E staining of skin tissue at the wound on day 12 in each group (Black arrow: cuticula, Green arrow: blood vessels, Blue arrows: inflammatory cells). **e** Masson staining of skin tissues at the wound on day 12 in each group. **f** Quantitative analysis of collagen deposition area in Masson staining (*n* = *4*). *P < 0.05, **P < 0.01, ***P < *0.001*

Results of the in vitro experiments showed that Tsg-THA&Fe40 hydrogel have good shape adaptation, tissue adhesion, antibacterial properties, and biocompatibility, thereby indicating that double-cross-linked gelatin-based hydrogels are ideal materials for traumatic wound dressings. To better verify the effect of the Tsg-THA&Fe40 hydrogel on wound healing, we constructed a full-thickness skin wound model in rats and compared the wound repair of the no treatment (group C), Comfeel^®^ Plus Transparent treatment (group P), and Tsg-THA&Fe40 hydrogel treatment (group E) groups. The effects of Tsg-THA&Fe hydrogel on wound healing were also evaluated. As shown in Fig. 6a, four days after healing, the wounds in groups C and P were significantly swollen. The wounds in Group C had obvious contaminants adhering to the wound surface. Group E had no swelling and fewer contaminants than those in groups C and P. The results showed that the Tsg-THA&Fe hydrogel could eliminate wound inflammation, reduce wound swelling, and effectively prevent the invasion of pollutants into the wound.

Wound closure marks were followed and statistically analyzed using digital photographs of the wounds and quantitative displays of closure rates (Fig. 6b, c). Group E had a smaller wound area than groups C and P, on day 4. On day 8, the wound area was significantly reduced in all groups (*P* < *0.01*), and group E was almost completely closed, which was a better outcome than those in groups C and P. On day 12, the healing rates of the groups C and P were 90% and 91%, respectively, whereas the E group had completely healed. On day 12, epithelial formation in group E was significantly better than that in group C and P (P < 0.001) (Additional file 1: Fig. S6). The results showed that the Tsg-THA&Fe hydrogel was better than the hydrocolloid dressing (Comfeel^®^ Plus Transparent), in terms of improving wound healing quality and shortening the wound healing time.

### Skin histological analysis

H&E and Masson staining were applied to further illustrate the effect of hydrogels on wound healing. The results of H&E staining of wound skin tissue showed that group E had reduced inflammatory infiltration, improved epithelial transformation, enhanced neovascularization, and increased migration of fibroblasts on day 12, as compared to groups C and P (Fig. 6d). In addition, Masson staining results (Fig. 6e, f), which can reflect collagen deposition in the process of wound healing, were shown. On day 12, compared with group C, collagen deposition was positively and significantly increased in group E (*P* < *0.001*) and collagen deposition was significantly increased in group P (*P* < *0.01*). These results suggested that the Tsg-THA&Fe hydrogel can improve wound healing by inhibiting inflammatory infiltration and promoting epidermal proliferation, angiogenesis, and collagen deposition.

### Expression of inflammatory factors in skin tissues

**Fig. 7:**
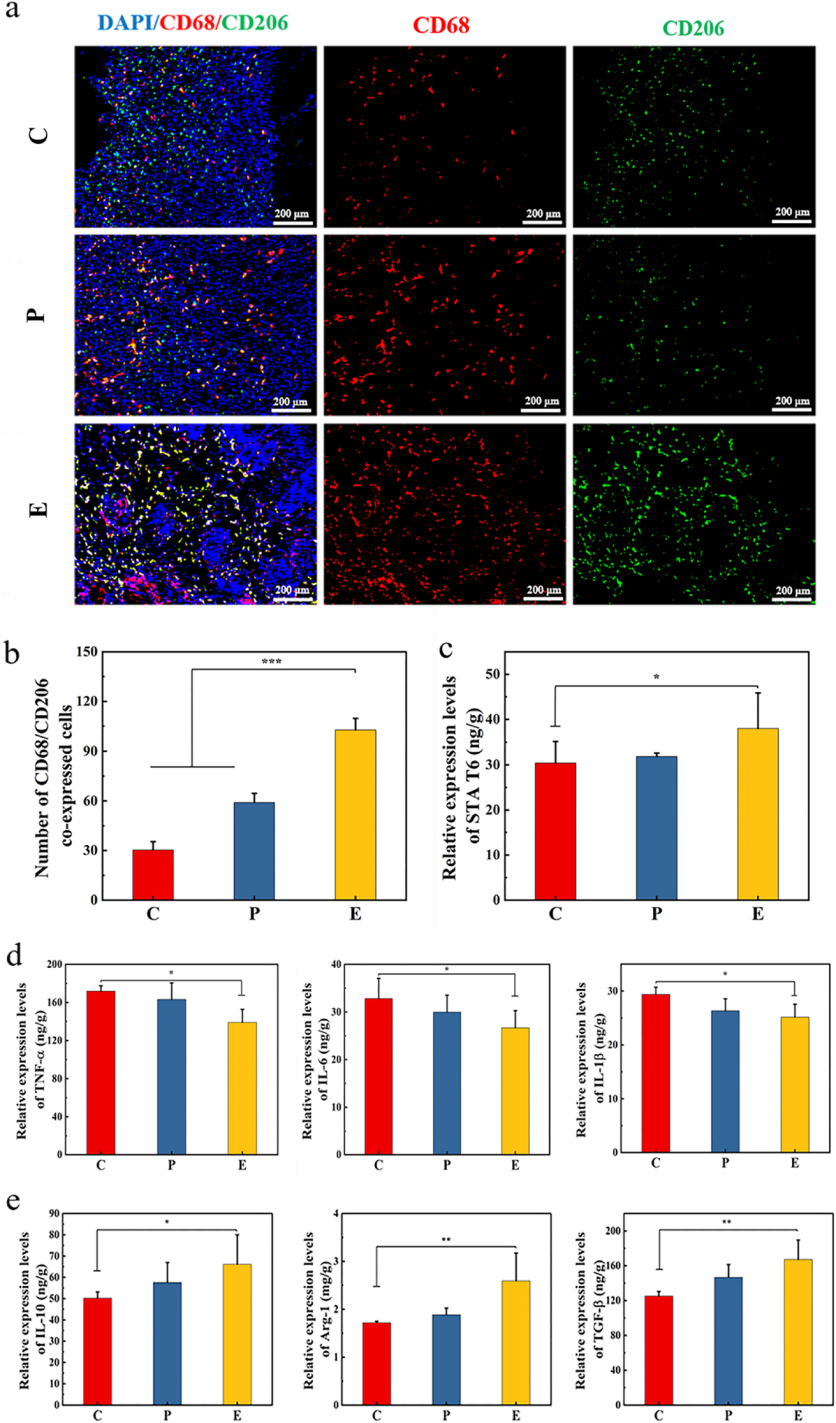

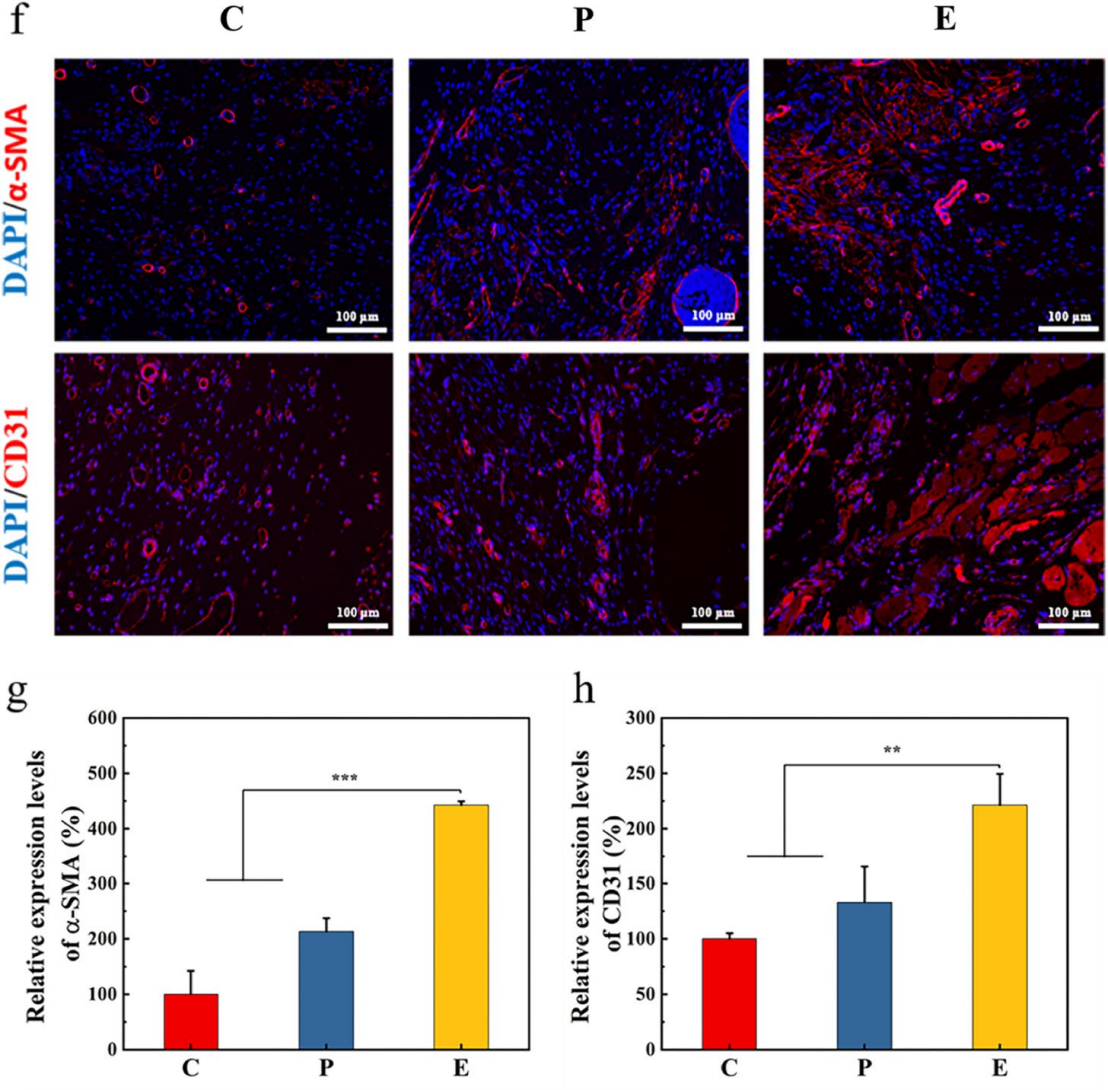
Hydrogel promotes wound healing by modulating the inflammatory response. **a** Immunofluorescence staining images of the pan-macrophage marker CD68 (red) and M2 macrophage marker CD206 (green) in the group C, group P and group E on day 8. **b** The number of CD68 and CD206 co-expressed cells in group C, group P and group E on day 8 (*n* = *4*, ***P < *0.001*). **c** The expression of signal transducer and activators of transcription 6 (STA T6) in wound of group C, P and E on day 8 (*n* = 4, *P < *0.05*). **d** The expression of tumor necrosis factor-alpha (TNF-α), interleukin-6 (IL-6), and interleukin-1β (IL-1β) in wound of group C, P and E on day 8 (*n* = *4*, *P < *0.05*). **e** The expression of interleukin-10 (IL-10), arginase-1 (Arg-1), and tumor growth factor-beta (TGF-β) in wound of group C, P and E on day 8 (*n* = *4*, *P < *0.05*). **f** Immunofluorescence staining images of α-SMA (red) and CD31 (red) in the wounds on day 12 in each group. Quantitative analysis of relative fluorescence intensity of **g** α-SMA and **h** CD31 stained skin tissue (*n* = *4*, **P < 0.01, ***P < *0.001*)

Macrophages play an important role in regulating inflammatory responses during wound healing. Activated macrophages can be divided into two phenotypes: M1 type (pro-inflammatory) and M2 type (anti-inflammatory). The M1 type mainly causes a strong inflammatory response in the body, while the M2 type reduces inflammation and remodels tissue by synthesizing anti-inflammatory factors and repair mediators [81–83]. Therefore, promoting M2 type polarization is an important approach for improving wound healing. Studies have shown that the Janus kinase I-signal transducer and activators of transcription 6 (JAKI-STAT6) pathway is related to the polarization of M2-type macrophages [84]. M2 macrophages usually use CD206 as a marker. The presence of M2 macrophages can be accurately assessed by means of co-expression of CD68 (a pan-macrophage marker) and CD206 [85, 86]. On day 8, immunofluorescence results showed that group E had a significantly increased number of M2 macrophages in the wound (*P* < *0.001*) (Fig. 7a, b), which was also confirmed by the significant upregulation of STAT6 protein expression in group E (*P* < *0.05*) (Fig. 7c). These results suggested that the Tsg-THA&Fe hydrogel can promote the conversion of macrophages to the M2 type during inflammation, thus effectively reducing the inflammatory response at the initial stage of wound healing. In addition, significant downregulation of tumor necrosis factor-alpha (TNF-α; *P* < *0.05*), interleukin-6 (IL-6; *P* < *0.05*), and interleukin-1β (IL-1β; *P* < *0.05*) as well as significant upregulation of interleukin-10 (IL-10; *P* < *0.05*), Arginase-1 (Arg-1; *P* < *0.01*), and tumor growth factor-beta (TGF-β; *P* < *0.01*) in group E, on day 8, further indicated regression of inflammation due to macrophage polarization (Fig. 7d, e).

According to previous studies, M2 macrophages produce the growth-promoting factors TGF-β and vascular endothelial growth factor (VEGF), to activate fibroblasts and stimulate angiogenesis [87]. On day 12, VEGF expression was the highest in Group E, which was significantly higher than that in group C (*P* < *0.05*) (Additional file 1: Fig. S7). Moreover, group E showed significantly improved expression of CD31 (a signaling molecule that regulates angiogenesis) (P < 0.001) andα-SMA (a marker of integrated fibroblast formation) (P < 0.01) (Fig. 7f–h). These results suggested that the Tsg-THA&Fe hydrogel had better pro-vascularization ability, accelerated tissue reconstruction, and improved wound healing. In addition, the Tsg-THA&Fe hydrogel displayed enhanced effects in promoting wound healing, with different advantages relative to hydrocolloid dressing (Comfeel^®^ Plus Transparent), which presents the possibility of clinical application of Tsg-THA&Fe hydrogel in wound healing.

### Wound microbiological analysis

The effect of hydrogels on in vivo bacterial inhibition is currently assessed by means of microbial culture of wound tissues for a number of microbial colonizations; this method, however, cannot describe the dynamic changes in the wound microbial composition [88]. Therefore, we used microbial genomics technology to sequence the 16S ribosomal DNA (16S rDNA) gene of wound microorganisms and evaluate the indicators of dynamic microbial diversity, microbial load and relative abundance of each group in the wound, thus providing a comprehensive description of the effect of Tsg-THA&Fe hydrogel on wound microorganisms [89, 90].

**Fig. 8:**
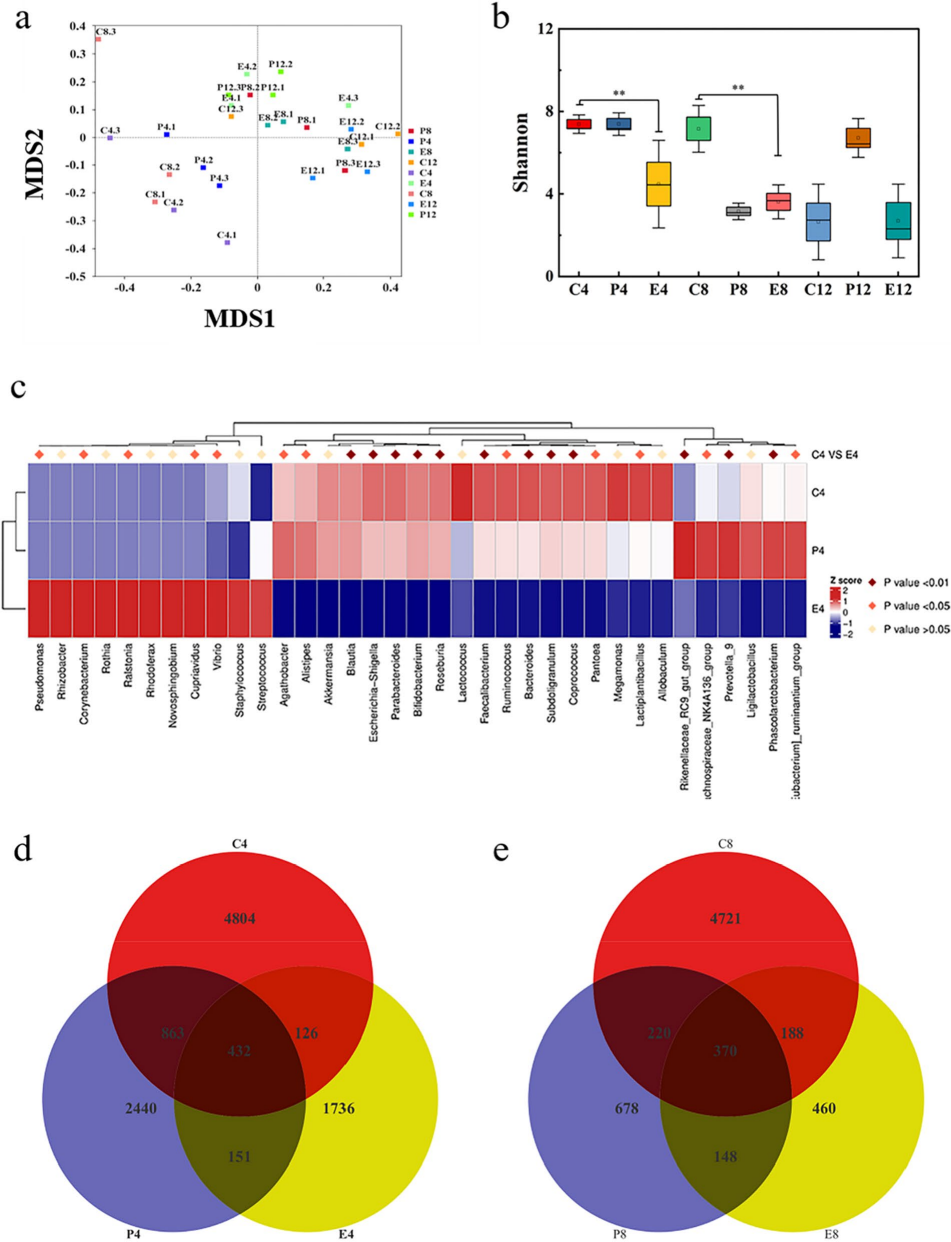
Bioinformatics analysis of wound microorganisms. In order to facilitate the comparison and analysis of data in each group, the labels of each group were defined as XY.n by Bioinformatics analysis. The X position mainly represented the untreated group (group C), Comfeel^®^ Plus Transparent treatment group (group P) and Tsg-THA&Fe40 hydrogel treatment group (group E). Y positions respectively represent different time points, mainly including day 4, 8 and 12. The n position represents the serial number of different samples in the group. **a** The results of Non-metric multidimensional scaling (NMDS) analysis based on OTU level using each group of samples are shown. each point in the figure indicates a sample, the distance between points indicates the degree of variation, and the samples of the same group are indicated using the same color. when Stress is less than 0.2, it indicates that NMDS can accurately reflect the degree of variation among samples. **b** The Alpha Diversity analysis index Shannon was counted for different samples at a 97% consistency threshold, and Shannon is the total number of taxa in the samples and their percentage. The higher the community diversity and the more evenly spread the species, the greater the Shannon index (*n* = 3, *P < 0.05, **P < *0.01*). **c** On day 4, Metastat heat map analysis for groups C4. The corresponding values in the heat map are the z-values obtained by normalizing the species in each row, and the z-values for samples in each classification are obtained by dividing the difference between the relative abundance of samples in that classification and the average relative abundance of all samples in that classification by the standard deviation of all samples in that classification. **d** and **e** The common and unique the operational taxonomic unit (OUT) among different groups are analyzed and plotted as a Venn Graph, where each circle represents a sample (group), and the number of overlapping circles represents the number of common OTU among samples (groups), and the number of numbers without overlapping circles represents the number of unique OTU of samples (groups)

The bacterial diversity of the untreated (group C), Comfeel^®^ Plus Transparent treatment (group P), and Tsg-THA&Fe40 hydrogel treatment groups on days 4, 8, and 12 was analyzed by means of 16S rDNA high-throughput sequencing. In the microbial informatics analysis, to facilitate the comparative analysis of the data of each group, the label of each group was defined as XY.n. The X-position mainly represents the untreated (group C), Comfeel^®^ Plus transparent treatment (group P), and Tsg-THA&Fe40 hydrogel treatment (group E) groups. The Y-position represents different time-points, including days 4, 8, and 12. The n-position indicates the serial number of different samples in the group. Clustering analysis on days 4, 8, and 12 showed a clear difference between the groups (Additional file 1: Fig. S8a). Non-metric multidimensional scaling (NMDS) analysis between groups, based on the biological information contained in the samples, showed differences between samples and complete separation of the biograms (Fig. 8a) [56]. On day 8, the Shannon index further showed a significant difference in abundance and homogeneity between the blank and hydrogel groups (Fig. 8b) (*P* < *0.01*). This indicated that the Tsg-THA&Fe hydrogel established a tight physical barrier from the wound, and the Tsg-THA&Fe hydrogel secured dynamic isolation of the wound from the external environment, by using its good adaptive performance as well as mechanical and adhesion properties.

We further analyzed the changes in microbial abundance in the wounds of each group, based on the species annotation results. As shown in the species abundance histograms at the genus and phylum levels (Additional file 1: Fig. S8b, c), the relative abundance of microorganisms differed over time and at different levels, in groups C, P, and E. Species with significant differences between groups C and E at the genus level on day 4 were studied using the Metastat heatmap (Fig. 8c). The relative abundances of five microorganisms in group C, including Pseudomonas, Corynebacterium, Lalstonia, Culex, and Vibrio, were significantly lower than those in group E, at the genus level (*P* < *0.05*). The relative abundance of 19 microorganisms, including *Agathobacter* (*P* < *0.05*), *Alistipes* (*P* < *0.05*), *Ruminococcus* (*P* < *0.05*), *Pantoea* (*P* < *0.05*), *Lactiplantibacillus* (*P* < *0.05*), *Lachnospiraceae_NK4A136_group* (*P* < *0.05*), *Eubacterium_ruminantium_group* (*P* < *0.05*), *Blautia* (*P* < *0.01*), *Escherichia−Shigella* (*P* < *0.01*), *Parabacteroides* (*P* < *0.01*), *Bifidobacterium* (*P* < *0.01*), *Roseburia* (*P* < *0.01*), *Faecalibacterium* (*P* < *0.01*), *Bacteroides* (*P* < *0.01*), *Subdoligranulum* (*P* < *0.01*), *Coprococcus* (*P* < *0.01*), *Rikenellaceae_RC9_gut_group* (*P* < *0.01*), *Prevotella_9* (*P* < *0.01*), and *Phascolarctobacterium* (*P* < *0.01*) were significantly lower in group E, as compared to those in group C. These data showed that the excellent biocompatibility and unique three-dimensional mesh structure of the Tsg-THA&Fe hydrogel enabled the hydrogel to interact benignly with the microbial system at the wound site, thereby significantly changing the relative abundance of some specific bacteria.

We quantified the wound microbial load in different groups for each period, based on the operational taxonomic unit (OUT) results (Fig. 8d, e). The obvious decrease in the number of bacteria in group E further validated the excellent antibacterial effect of the Tsg-THA&Fe hydrogel, which is produced by the synergistic effect of Schiff base compounds and Fe^3+^, indicating that the Tsg-THA&Fe hydrogel can isolate the entry of microorganisms into the external environment and reduce the massive colonization of microorganisms in the internal environment of the wound, to prevent wound infection [91].

The above data analysis shows that the Tsg-THA&Fe hydrogel changed the diversity of wound microorganisms, stabilized the wound microbial environment, and reduced the colonization of wound microorganisms. To better understand whether the effect of hydrogel on improving the wound healing was associated with its effect on microorganisms at the wound site, we investigated the relationship between changes in microbial dynamics and innate immunity in wounds. Toll-like receptors (TLRs) are an evolutionarily conserved set of pattern recognition receptors that recognize metabolites generated by bacteria, viruses, and other pathogens [92]. Toll-like receptor 2 (TLR2) and Toll-like receptor 4 (TLR4) stress in response to microbial contamination triggers intracellular signals, such as Nuclear Factor-Kappa B (NF-kB) [93]. Some specific bacteria can produce LPS, which is associated with wound immune responses, to induce the production of pro-inflammatory factors (such as IL-6 and TNF-α) and inhibit IL-10 secretion [94].

**Fig. 9:**
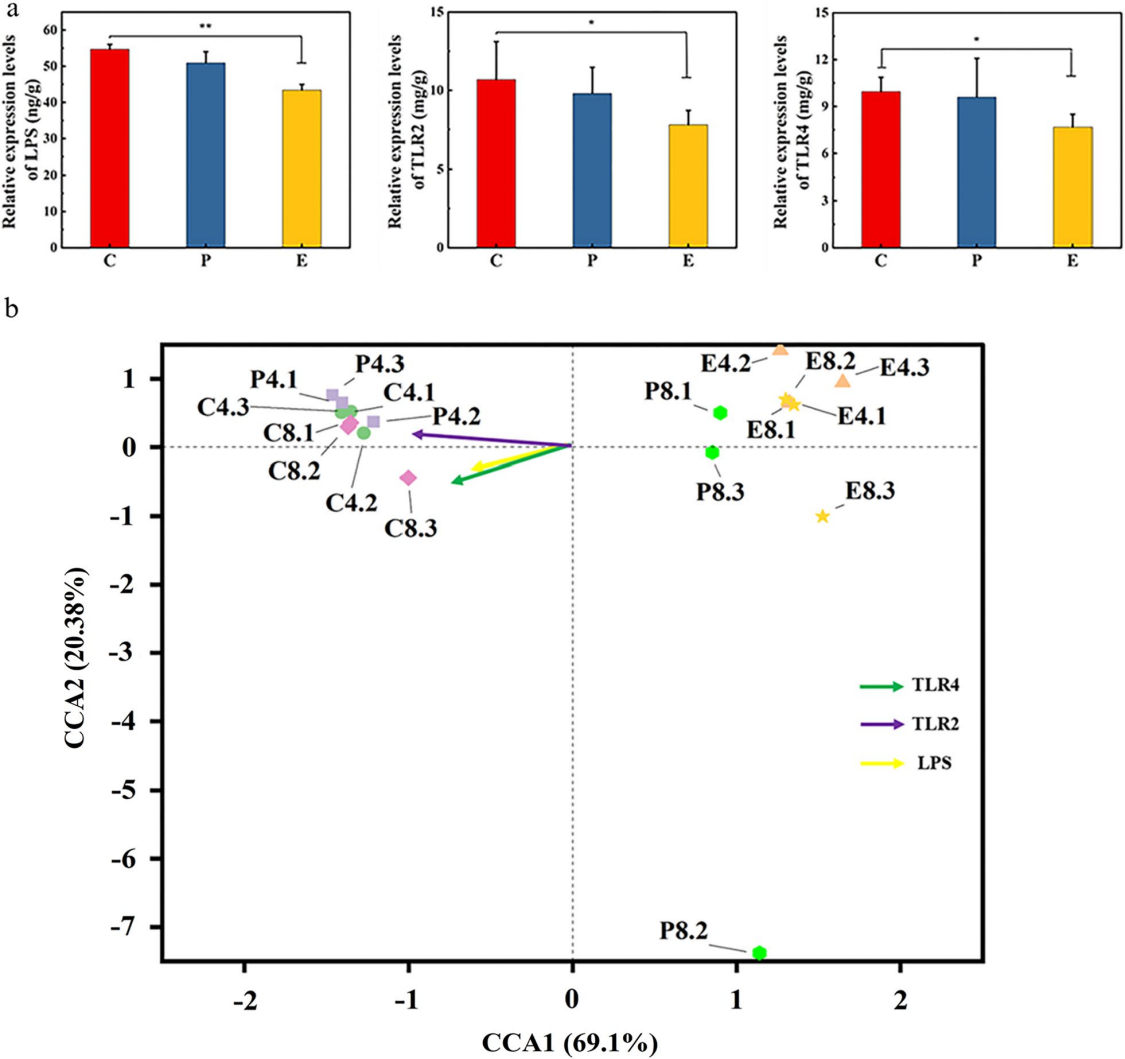
Relationship between wound microorganisms and innate immunity. **a** The expression levels of wound inflammatory factors lipopolysaccharide (LPS), toll-like receptor 2 (TLR2) and toll-like receptor 4 (TLR4) in untreated wound group (group C), Comfeel Plus Transparent treatment group (group P) and Tsg-THA&Fe40 hydrogel treatment group on day 8 (*n* = 3, *P < 0.05, **P < *0.01*). **b** The canonical correspondence analysis (CCA) was used mainly to reflect the relationship between the groups' colony-based distances and environmental factors. The horizontal coordinate indicates the first principal component, and the percentage indicates the contribution of the first principal component to the sample variation; the vertical coordinate indicates the second principal component, and the percentage indicates the contribution of the second principal component to the sample variation; each point in the graph represents one sample. The length of the arrow line represents the correlation between an environmental factor and the distribution of communities and species, the longer the line, the higher the correlation, and vice versa, the lower the correlation. The angle between the arrow line and the ranking axis represents the correlation between an environmental factor and the ranking axis, the smaller the angle, the higher the correlation, and vice versa

Group E showed a significant decrease in LPS (*P* < *0.01*), TLR2 (*P* < *0.05*), and TLR4 (*P* < *0.05*) levels (Fig. 9a), as compared to those in group C. The correlation between wound environmental factors (LPS, TLR2, and TLR4) and wound microorganisms was verified using the canonical correspondence analysis (CCA) with 16S rDNA high-throughput sequencing (Fig. 9b) [57]. The envfit function test showed a significant effect between the four environmental factors and the microbes (Additional file 1: Table S2). These results suggested that the significant reduction in LPS, TLR2, and TLR4 levels in group E may be directly related to the alteration of wound microorganism diversity and reduction of bacterial colonization [95, 96]. Tsg-THA&Fe hydrogel altered the microbial load, modulated changes in inflammatory factors, and promoted wound healing in traumatic wounds.

### In vivo safety assessment of hydrogels

**Fig. 10:**
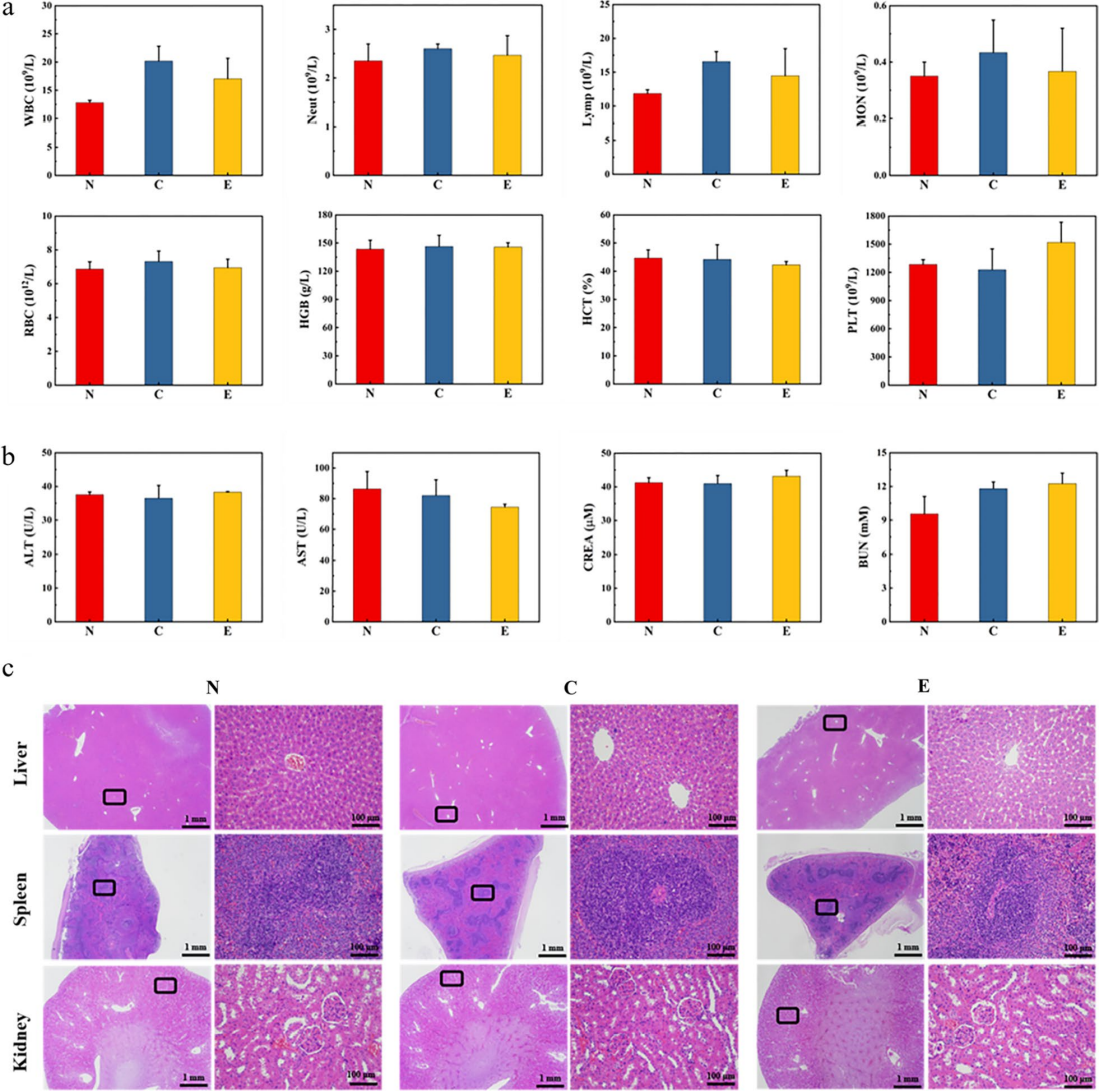
In vivo toxicity analysis was performed in the normal (group N), untreated (group C), and Tsg-THA&Fe40 hydrogel (group E) groups. **a** Routine blood tests of rats in groups N, C, and E, after 12 days, including assessment of white blood cells (WBC), neutrophils (Neut), lymphocyte count (Lymph), monocyte count (Mon), red blood cells (RBC), hemoglobin (HGB), red blood cell cumulative pressure (HCT) and platelets (PLT) (*n* = *4*). **b** Measurement of serum liver and kidney function indices in each group of rats, including the assessment of glutamic aminotransferase (ALT), glutamic oxalacetic aminotransferase (AST), serum creatinine (CREA), and urea nitrogen (BUN) levels (*n* = *4*). **c** Sections of the liver, spleen, and kidney of each group of rats were stained with H&E staining for pathological analysis

Finally, we tested the routine blood, liver, and kidney functions, to assess the safety of hydrogel application in vivo. The levels of white blood cells (WBCs), neutrophils (Grans), lymphocytes (Lymphs), and monocytes (Mons) were higher in each group compared to those in group N, which may be related to the protective immune response provoked by trauma (Fig. 10a) [97]. There were no significant changes in the levels of red blood cells (RBCs), hemoglobin (HGB), red blood cell extrusion (HCT), and platelets (PLTs), as compared to those in group N (Fig. 10a). Moreover, the Tsg-THA&Fe hydrogel treatment did not cause any significant changes in liver and kidney function indicators, such as glutamic aminotransferase (ALT), glutamic aminotransferase (AST), creatinine (Cre), and blood urea nitrogen (BUN) (Fig. 10b). In addition, H&E staining of the major organs of rats, such as the liver, kidney, and spleen (Fig. 10c), showed no histopathological alterations (necrosis and cytoarchitecture) in group E. These results indicated that the Tsg-THA&Fe hydrogel exhibited a good safety profile for wound treatment, without any obvious side effects on blood routine and organ function in rats, and did not cause metabolic abnormalities.

## Conclusions

In summary, the Tsg-THA&Fe hydrogel exhibited injectable and autonomous healing properties, excellent antibacterial properties, and good biocompatibility, to effectively accelerate wound healing. The materialized double-cross-linked network allowed the hydrogel to gel rapidly, possessed mechanical properties, and enhanced the adhesion performance. The Tsg-THA&Fe hydrogel regulated the positive conversion of macrophages to the M2 type, by reducing the expression of the pro-inflammatory cytokines TNF-α, IL-6, IL-8, and IL-1β, and upregulating the expression of IL-10, Arg-1, and TGF-β, to slow down wound inflammation, which in turn increased the expression of VEGF, CD31, and α-SMA, to improve wound healing. Wound microbiological analysis showed that Tsg-THA&Fe hydrogel altered the wound microbial load, modulated the expression of immune factors, such as LPS, TLR2, and TLR4, and reduced the possibility of wound transformation, suggesting the possibility of Tsg-THA&Fe hydrogel application in chronic wounds. Therefore, the Tsg-THA&Fe hydrogel exhibited excellent improvement in wound healing and has good prospects for wound dressing application.

## Supplementary Information

**Additional file 1: Figure S1.** **a** Raman spectrum of the THA&Fe powder. **b** Compression-strain curves of the Tsg-THA&Fe hydrogel in the strain range of 0–90%. **Table S1**. Sample composition and gel time of Tsg-THA&Fe hydrogel. **Figure S2**. **a** The average pore size of the hydrogels. **b** Injectable properties of the Tsg-THA&Fe hydrogel in PBS (37 °C, pH 7.4). **Figure S3** Photographs of the Tsg-THA&Fe hydrogel hemolytic activity assay. **Figure S4 a** Adhesion of the Tsg-THA&Fe40 hydrogel to human finger joints. **b** A demonstration of the adhesion of the Tsg-THA&Fe40 hydrogel to pig skin, with twisting effect. **c** Demonstration of the adhesion of the Tsg-THA&Fe40 hydrogel to rat heart, liver, spleen, and kidney. **Figure S5 a** and **b** the hemostatic effect of the Tsg-THA&Fe40 hydrogel was evaluated in the rat broken tail and liver hemorrhage models. **Figure S6** The epidermal thickness of different wounds on day 12 was quantitatively analyzed (*n* = 4, *p < 0.05, **p < 0.01, ***p < *0.001*). **Figure S7** Expression of the cytokine vascular endothelial growth factor (VEGF) at the wound on day 12 after different treatments (*n* = *4*, *P < *0.05*). **Figure S8 a** Unweighted Pair-group Method with Arithmetic Means (UPGMA) clustering analysis with Weighted UniFrac distance matrix for each group of samples at different periods, and integration of the clustering results with the relative abundance of the species at the phylum level for each sample. The UPGMA clustering tree structure is on the left, while the distribution of the relative abundance of species at the phylum level for each sample on the right. **b** and **c** The Top 10 species in each group in terms of maximum abundance at the phylum and genus taxonomic levels were analyzed to generate a cumulative bar graph of the species relative abundance, to visualize the species with a higher relative abundance and their proportions at different taxonomic levels for each sample, with the horizontal coordinate (Sample Name) being the group name and the vertical coordinate (Relative Abundance) indicating the relative abundance; Others indicates the sum of the relative abundance of all the other phyla in the graph, except these 10 phyla. **Table S2**. CCA envfit table gives the significance analysis results of the environmental factors, such as lipopolysaccharide (LPS), toll-like receptor 2 (TLR2) and toll-like receptor 4 (TLR4). CCA1 and CCA2 are the cosines of the angle between the arrow and the ranking axis of the environmental factor, which indicates the correlation between the environmental factor and the ranking axis. r^2^ indicates the coefficient of determination of the environmental factor on the species distribution, and the smaller r^2^ means that the environmental factor has less influence on the species distribution. P indicates the significance test of the correlation.

## Data Availability

All data generated or analyzed during this study are included in this published article (and its additional files).
