# Correction: A physicochemical double-cross-linked gelatin hydrogel with enhanced antibacterial and anti-inflammatory capabilities for improving wound healing

**DOI:** 10.1186/s12951-023-02029-4

**Published:** 2023-08-08

**Authors:** Yapeng Lu, Meihui Zhao, Ye Peng, Sizhe He, Xiaopeng Zhu, Chao Hu, Guanghua Xia, Tao Zuo, Xueying Zhang, Yonghuan Yun, Weimin Zhang, Xuanri Shen

**Affiliations:** 1 Hainan Engineering Research Center of Aquatic Resources Efficient Utilization in South China Sea, Key Laboratory of Food Nutrition and Functional Food of Hainan Province, Key Laboratory of Seafood Processing of Haikou, College of Food Science and Technology, Hainan University, Hainan, 570228 China; 2 Faculty of Medicine, Macau University of Science and Technology, Taipa, Macao SAR China; 3 Collaborative Innovation Center of Provincial and Ministerial Co-Construction for Marine Food Deep Processing, Dalian Polytechnic University, Dalian, 116034 China; 4 Guangdong Institute of Gastroenterology, The Sixth Affiliated Hospital of Sun Yat-sen University, Sun Yat-sen University, Guangzhou, 510000 China

**Correction: Journal of Nanobiotechnology (2022) 20: 426** 10.1186/s12951-022-01634-z

Following publication of the original article [1], the authors identified an image duplication problem in Fig. 3b. The corrected Fig. 3 are given below.

**Fig. 3:**
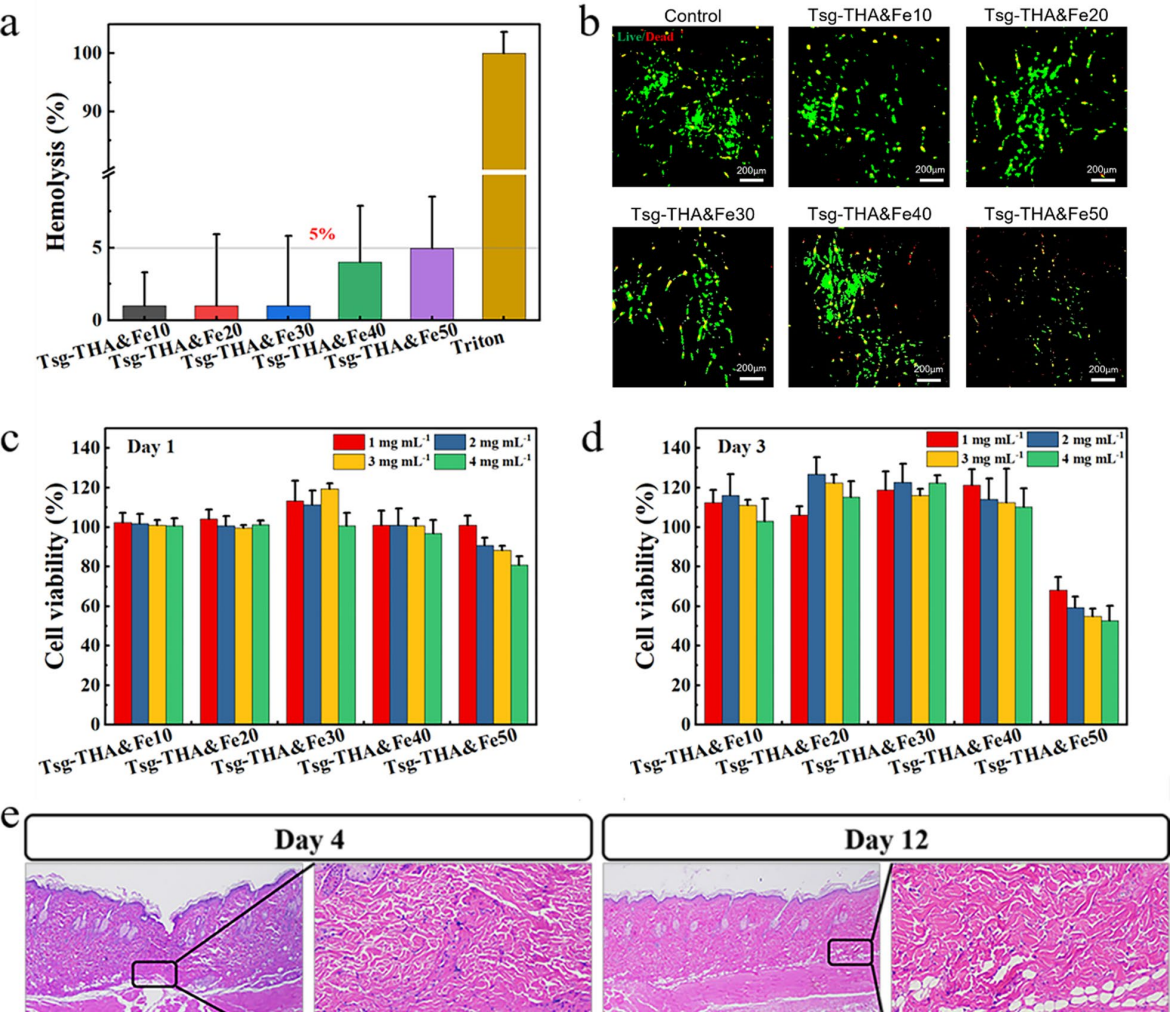
Biocompatibility of the Tsg-THA&Fe hydrogel. **a** Hemolysis assay of Tsg-THA&Fe hydrogel (*n* = *3*). **b** Cell staining of NIH-3T3 cells cultured in the Tsg-THA&Fe hydrogel for 3 days. Survival rate of NIH-3T3 cells cultured in each group at different concentrations of hydrogel leachate for 1 **c** and 3 **d** days (*n* = *6*). **e** Hematoxylin–eosin (H&E) staining of skin tissue implanted subcutaneously with Tsg-THA&Fe40 hydrogel, the box shows the approximate location of hydrogel implantation (*n* = *5*)

In addition, in the Live/Dead cell staining experiments, the culture time of cells and hydrogels should be revised to "3 days".

The author apologizes for any inconvenience caused.

The original article [1] has been revised.
