# Supplementary material for: A physicochemical double-cross-linked gelatin hydrogel with enhanced antibacterial and anti-inflammatory capabilities for improving wound healing
Source: J Nanobiotechnology. 2022 Sep 24;20:426. doi: 10.1186/s12951-022-01634-z (PMC9509571; doi:10.1186/s12951-022-01634-z)
Supplement: Supplementary file 1 — Additional file 1: Figure S1. a Raman spectrum of the THA&Fe powder. b Compression-strain curves of the Tsg-THA&Fe hydrogel in the strain range of 0–90%. Table S1. Sample composition and gel time of Tsg-THA&Fe hydrogel. Figure S2. a The average pore size of the hydrogels. b Injectable properties of the Tsg-THA&Fe hydrogel in PBS (37 °C, pH 7.4). Figure S3 Photographs of the Tsg-THA&Fe hydrogel hemolytic activity assay. Figure S4 a Adhesion of the Tsg-THA&Fe40 hydrogel to human finger joints. b A demonstration of the adhesion of the Tsg-THA&Fe40 hydrogel to pig skin, with twisting effect. c Demonstration of the adhesion of the Tsg-THA&Fe40 hydrogel to rat heart, liver, spleen, and kidney. Figure S5 a and b the hemostatic effect of the Tsg-THA&Fe40 hydrogel was evaluated in the rat broken tail and liver hemorrhage models. Figure S6 The epidermal thickness of different wounds on day 12 was quantitatively analyzed (n = 4, *p < 0.05, **p < 0.01, ***p < 0.001). Figure S7 Expression of the cytokine vascular endothelial growth factor (VEGF) at the wound on day 12 after different treatments (n = 4, *P < 0.05). Figure S8 a Unweighted Pair-group Method with Arithmetic Means (UPGMA) clustering analysis with Weighted UniFrac distance matrix for each group of samples at different periods, and integration of the clustering results with the relative abundance of the species at the phylum level for each sample. The UPGMA clustering tree structure is on the left, while the distribution of the relative abundance of species at the phylum level for each sample on the right. b and c The Top 10 species in each group in terms of maximum abundance at the phylum and genus taxonomic levels were analyzed to generate a cumulative bar graph of the species relative abundance, to visualize the species with a higher relative abundance and their proportions at different taxonomic levels for each sample, with the horizontal coordinate (Sample Name) being the group name and the vertical coordinate (Re [file 12951_2022_1634_MOESM1_ESM.docx]

**Additional file 1**


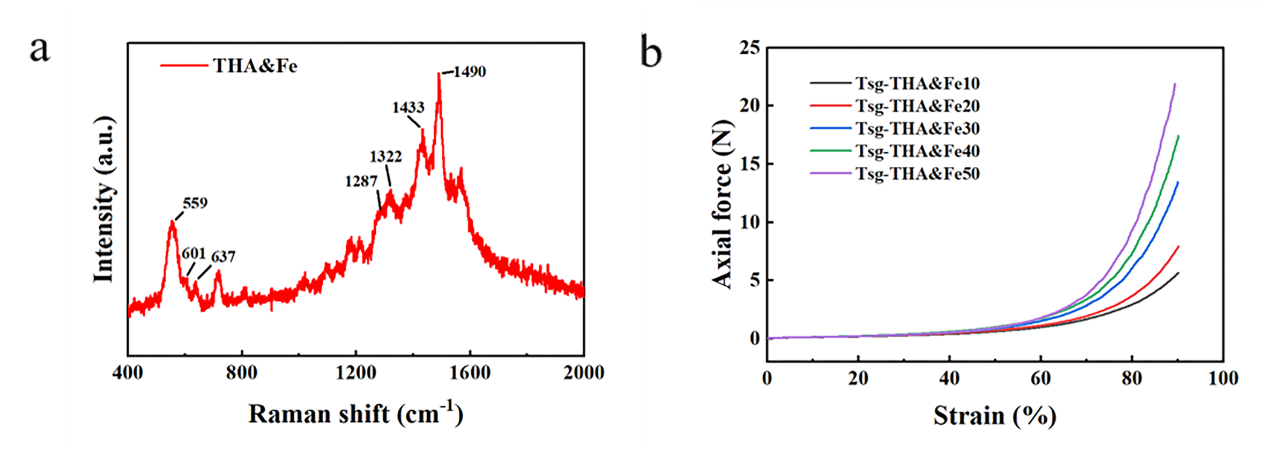


**Fig. S1**. **a** Raman spectrum of the THA&Fe powder. **b** Compression-strain curves of the Tsg-THA&Fe hydrogel in the strain range of 0%–90%.

**Table S1**. Sample composition and gel time of Tsg-THA&Fe hydrogel.

| **Sample** | **5% Tsg [mL]** | **THA&Fe [μL]** | **n(CHO):(NH_2_)** | **Gelation time [min]** |
| --- | --- | --- | --- | --- |
| **Tsg-THA&Fe50** | **3** | **50** | **0.8:1** | **5±1** |
| **Tsg-THA&Fe40** | **3** | **40** | **0.64:1** | **6±3** |
| **Tsg-THA&Fe30** | **3** | **30** | **0.48:1** | **12±2** |
| **Tsg-THA&Fe20** | **3** | **20** | **0.32:1** | **23±5** |
| **Tsg-THA&Fe10** | **3** | **10** | **0.16:1** | **30±2** |


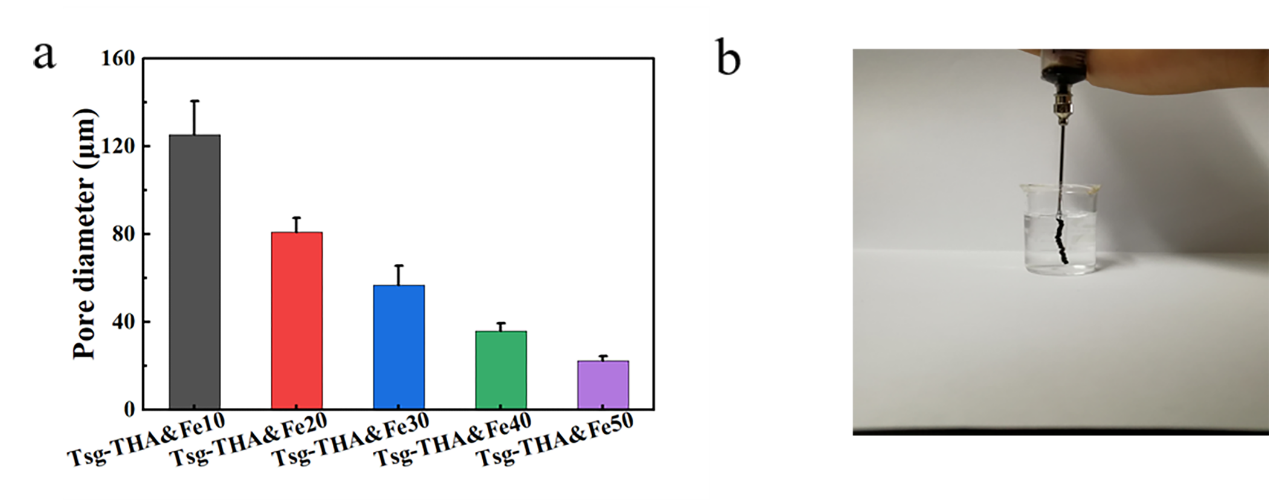


**Figure S2**. **a** The average pore size of the hydrogels. **b** Injectable properties of the Tsg-THA&Fe hydrogel in PBS (37°C, pH 7.4).


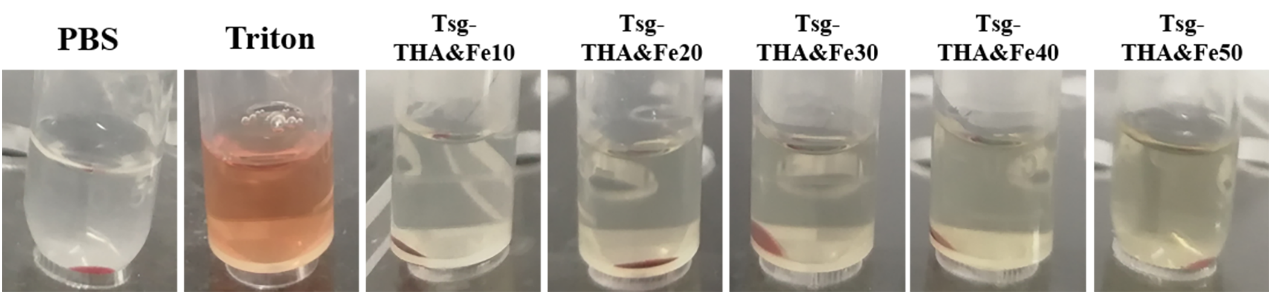
**Fig. S3** Photographs of the Tsg-THA&Fe hydrogel hemolytic activity assay.


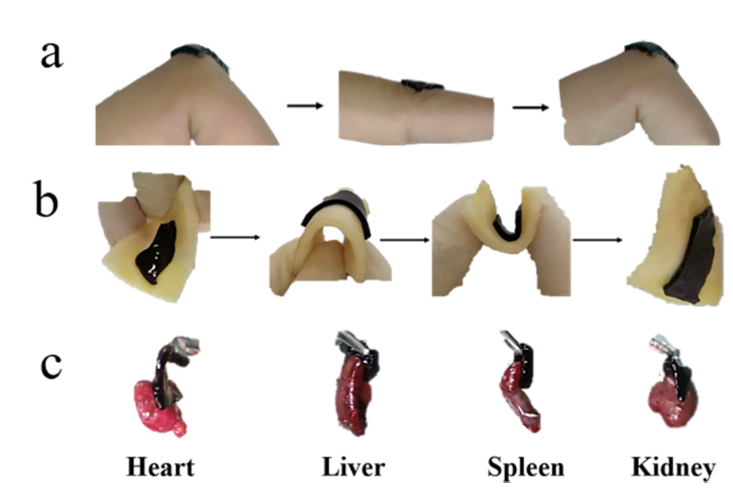


**Fig. S4** **a** Adhesion of the Tsg-THA&Fe40 hydrogel to human finger joints. **b** A demonstration of the adhesion of the Tsg-THA&Fe40 hydrogel to pig skin, with twisting effect. **c** Demonstration of the adhesion of the Tsg-THA&Fe40 hydrogel to rat heart, liver, spleen, and kidney.


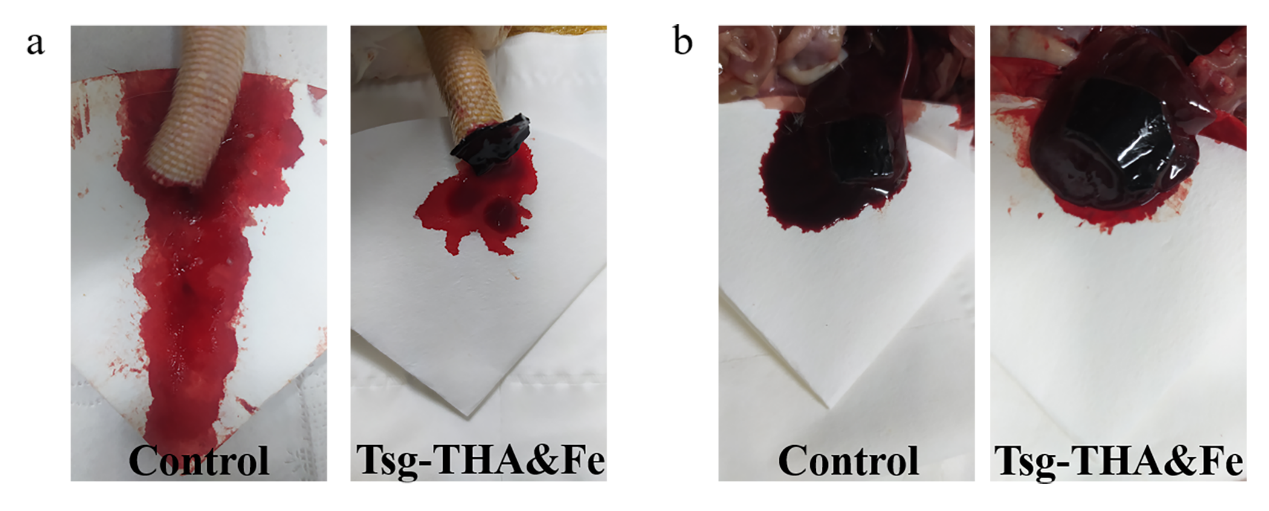


**Fig. S5** **a** and **b** the hemostatic effect of the Tsg-THA&Fe40 hydrogel was evaluated in the rat broken tail and liver hemorrhage models.


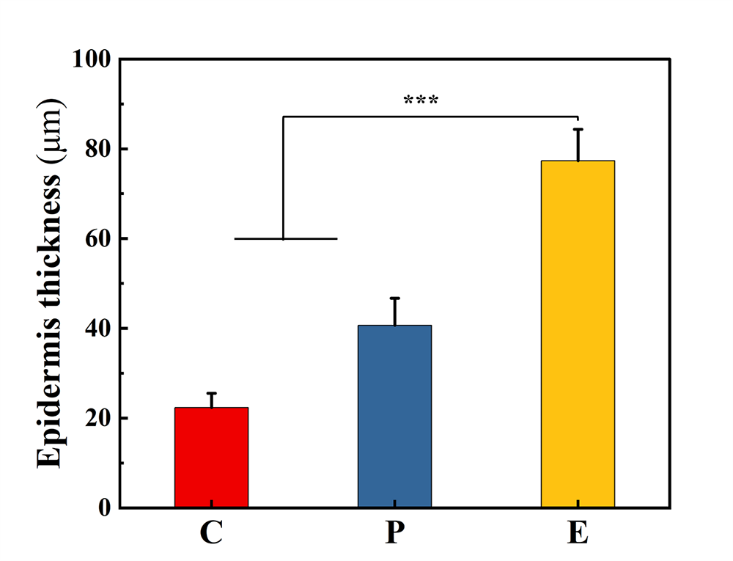


**Fig. S6** The epidermal thickness of different wounds on day 12 was quantitatively analyzed (*n = 4, *p < 0.05, **p < 0.01, ***p < 0.001*).


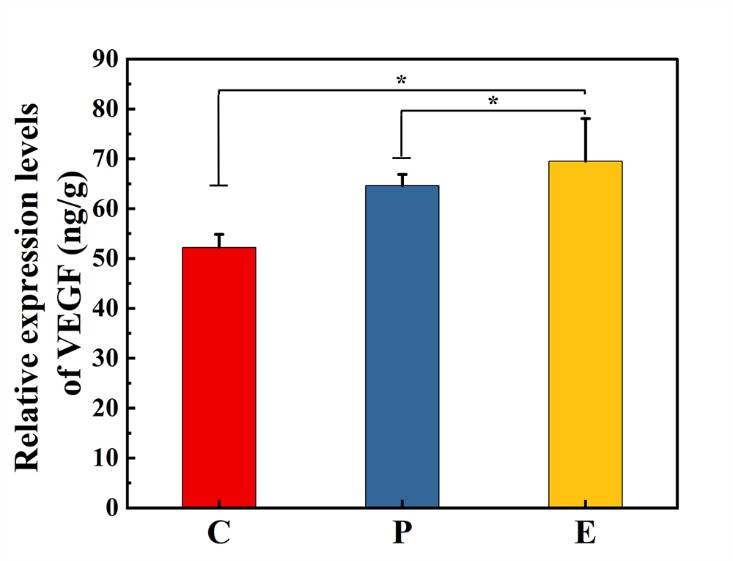


**Fig. S7** Expression of the cytokine vascular endothelial growth factor (VEGF) at the wound on day 12 after different treatments (*n*=*4*, **P<0.05*).


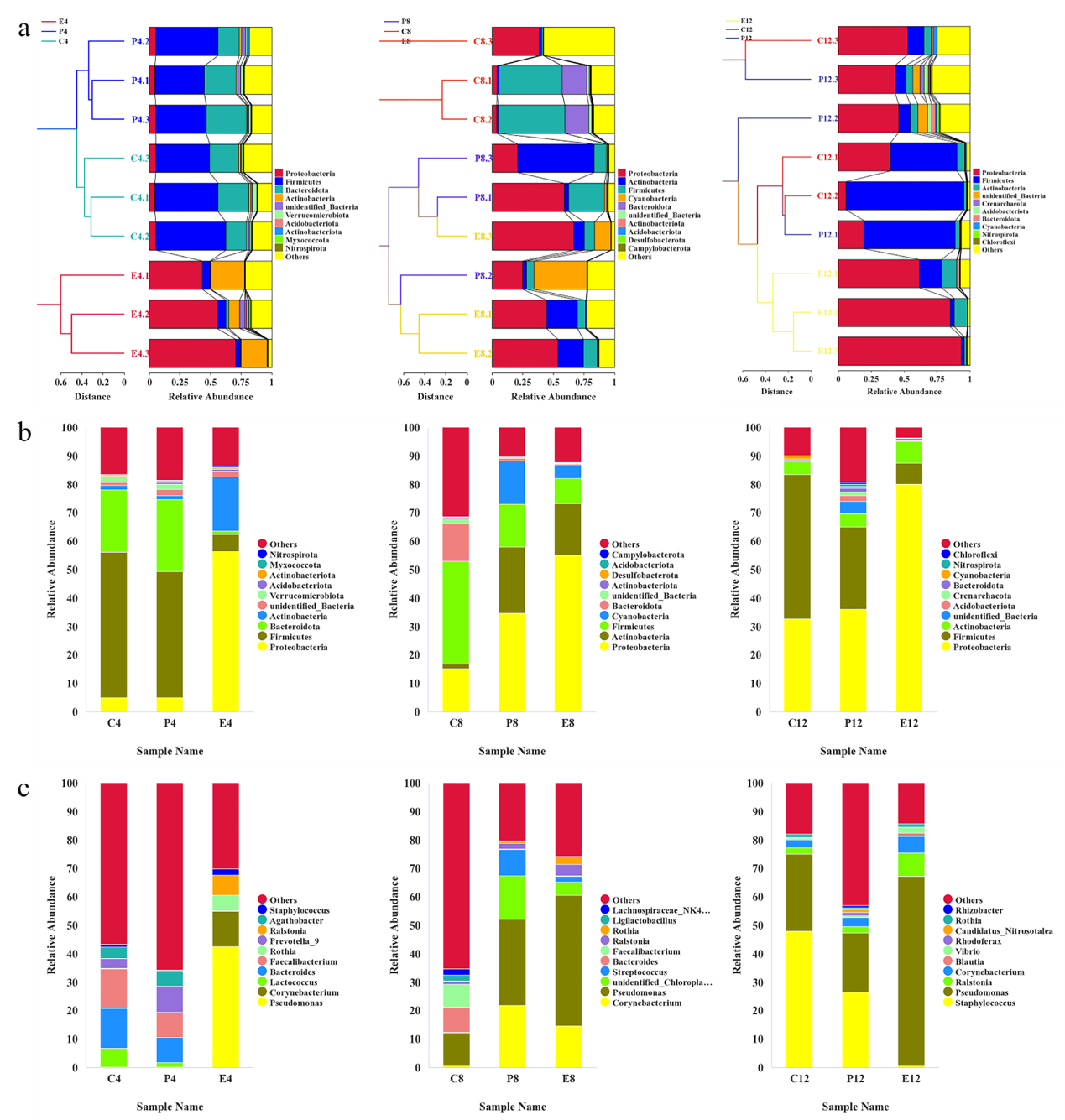


**Fig. S8** **a** Unweighted Pair-group Method with Arithmetic Means (UPGMA) clustering analysis with Weighted UniFrac distance matrix for each group of samples at different periods, and integration of the clustering results with the relative abundance of the species at the phylum level for each sample. The UPGMA clustering tree structure is on the left, while the distribution of the relative abundance of species at the phylum level for each sample on the right. **b** and **c** The Top 10 species in each group in terms of maximum abundance at the phylum and genus taxonomic levels were analyzed to generate a cumulative bar graph of the species relative abundance, to visualize the species with a higher relative abundance and their proportions at different taxonomic levels for each sample, with the horizontal coordinate (Sample Name) being the group name and the vertical coordinate (Relative Abundance) indicating the relative abundance; Others indicates the sum of the relative abundance of all the other phyla in the graph, except these 10 phyla.

**Table S2**. CCA envfit table gives the significance analysis results of the environmental factors, such as lipopolysaccharide (LPS), toll-like receptor 2 (TLR2) and toll-like receptor 4 (TLR4). CCA1 and CCA2 are the cosines of the angle between the arrow and the ranking axis of the environmental factor, which indicates the correlation between the environmental factor and the ranking axis. r^2^ indicates the coefficient of determination of the environmental factor on the species distribution, and the smaller r^2^ means that the environmental factor has less influence on the species distribution. P indicates the significance test of the correlation.

|  | **CCA1** | **CCA2** | **r^2^** | **P** |
| --- | --- | --- | --- | --- |
| **LPS** | **-0.56563** | **-0.32124** | **0.25781** | **0.0079** |
| **TLR2** | **-0.93086** | **0.18306** | **0.51461** | **0.0024** |
| **TLR4** | **-0.69896** | **-0.48874** | **0.43007** | **0.0104** |
